# Fine-scale adaptive divergence and population genetic structure of *Aedes aegypti* in Metropolitan Manila, Philippines

**DOI:** 10.1186/s13071-024-06300-x

**Published:** 2024-05-21

**Authors:** Atikah Fitria Muharromah, Thaddeus M. Carvajal, Maria Angenica F. Regilme, Kozo Watanabe

**Affiliations:** 1 Center for Marine Environmental Studies (CMES), Ehime University, Bunkyo-cho 3, Matsuyama, Ehime 7908577 Japan; 2 Graduate School of Science and Engineering, Ehime University, Bunkyo-cho 3, Matsuyama, Ehime 7908577 Japan; 3 Department of Tropical Biology, Faculty of Biology, Universitas Gadjah Mada, Yogyakarta, 55281 Indonesia; 4 Biological Control Research Unit, Center for Natural Sciences and Environmental Research, De La Salle University, 2401 Taft Avenue, 1004 Manila, Philippines

**Keywords:** *Aedesaegypti*, Dengue, ddRAD–Seq, SNPs

## Abstract

**Background:**

The adaptive divergence of *Aedes aegypti* populations to heterogeneous environments can be a driving force behind the recent expansion of their habitat distribution and outbreaks of dengue disease in urbanized areas. In this study, we investigated the population genomics of *Ae. aegypti* at a regional scale in Metropolitan Manila, Philippines.

**Methods:**

We used the Pool-Seq double digestion restriction-site association DNA sequencing (ddRAD-Seq) approach to generate a high number of single nucleotide polymorphisms (SNPs), with the aim to determine local adaptation and compare the population structure with 11 microsatellite markers. A total of 217 *Ae. aegypti* individuals from seven female and seven male populations collected from Metropolitan Manila were used in the assays.

**Results:**

We detected 65,473 SNPs across the populations, of which 76 were non-neutral SNPs. Of these non-neutral SNPs, the multivariate regression test associated 50 with eight landscape variables (e.g. open space, forest, etc.) and 29 with five climate variables (e.g. air temperature, humidity, etc.) (*P*-value range 0.005–0.045) in female and male populations separately. Male and female populations exhibited contrasting spatial divergence, with males exhibiting greater divergence than females, most likely reflecting the different dispersal abilities of male and female mosquitoes. In the comparative analysis of the same *Ae. aegypti* individuals, the pairwise *F*_ST_ values of 11 microsatellite markers were lower than those of the neutral SNPs, indicating that the neutral SNPs generated via pool ddRAD-Seq were more sensitive in terms of detecting genetic differences between populations at fine-spatial scales.

**Conclusions:**

Overall, our study demonstrates the utility of pool ddRAD-Seq for examining genetic differences in *Ae. aegypti* populations in areas at fine-spatial scales that could inform vector control programs such as *Wolbachia*-infected mosquito mass-release programs. This in turn would provide information on mosquito population dispersal patterns and the potential barriers to mosquito movement within and around the release area. In addition, the potential of environmental adaptability observed in *Ae. aegypti* could help population control efforts.

**Graphical Abstract:**

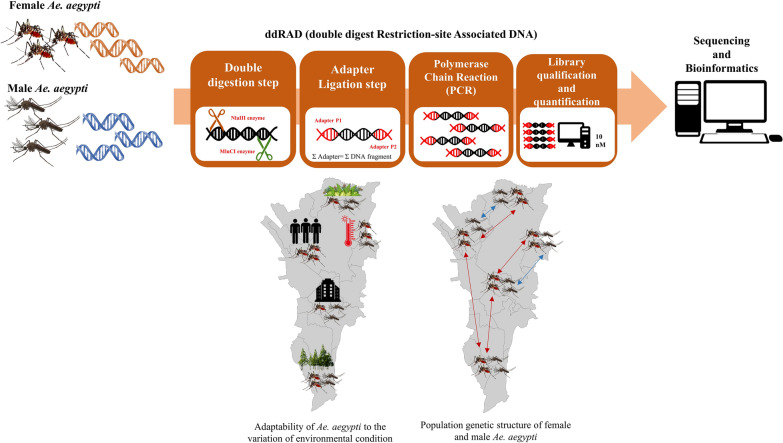

**Supplementary Information:**

The online version contains supplementary material available at 10.1186/s13071-024-06300-x.

## Background

*Aedes aegypti* is an important vector of mosquito-borne diseases, including dengue disease [1, 2]. In recent decades, the number of dengue disease cases has increased in urbanized areas, possibly owing to the recent habitat expansion of *Ae. aegypti* [3, 4]. This expansion suggests that *Ae. aegypti* possesses genetic adaptations to urban environments (e.g. human settlements). One study in Florida (USA) found that there was a higher abundance of *Ae. aegypti* in urban areas than in rural areas [5]. Thus, human populations in urban areas will be increasingly coming into contact with the *Ae. aegypti* mosquito, thereby increasing the risk of dengue transmission. In one study, the authors reported that a high number of female *Ae. aegypti* mosquitoes in urban/city areas was positively correlated with the number of dengue cases [6]. In addition to possessing a high adaptability to urban environments, *Ae. aegypti* possesses a high dispersal ability that may contribute to their niche expansion [1, 7]. Understanding the ecology of vectors with respect to their environmental adaptation and dispersal ability may allow researchers to predict the expansion of their habitat distribution under changing environmental conditions, such as changing landscape and weather conditions [7, 8]. An improved understanding of these factors can be obtained through population and landscape genomics approaches [9, 10].

Double digestion restriction-site association DNA sequencing (ddRAD-Seq) is a technique that facilitates genomic analysis by generating a high number of single nucleotide polymorphisms (SNPs) [11, 12]. Among the many SNPs generated, the few loci affected by directional selection should exhibit greater genetic differentiation than the neutral loci comprising the majority of the genome, whereas the few loci subject to balancing selection should exhibit lower genetic differentiation. These “outlier” loci can be identified as non-neutral loci through statistical methods. The environmental factors that cause natural selection can be estimated based on the correlation between non-neutral loci and environmental variables.

The investigation of the adaptive divergence of *Ae. aegypti* along an environmental gradient at a broad-scale environment has attracted the interest of researchers [13, 14]. Sherpa et al. [13] identified potential adaptive loci associated with human density and/or insecticide resistance at a continental scale, i.e. Africa and the Caribbean. A national-scale study in Panama revealed that *Ae. aegypti* populations were undergoing adaptive divergence along environmental gradients of temperature and vegetation [14]. However, adaptive divergence has not previously been examined at a fine-scale, for example, within a city, in which spatial genetic variance and environmental heterogeneity are usually low. Understanding the adaptive divergence of *Ae. aegypti* in a fine-scale area may help to reveal the recent selection of *Ae. aegypti* mosquitoes that is linked to environmental change [13, 14] and improve the accuracy of spatial forecast models of dengue vector populations when the local adaptation is occurring across the populations [14].

Neutral loci have been studied extensively to understand neutral evolutionary processes, including migration and genetic drift. SNPs generated in abundance through next-generation sequencing (NGS) via ddRAD-Seq are preferably used in population genetics studies because they allow the clear detection of population genetic structure, even at a fine-spatial scale [15]. Rašić et al. compared the ability of microsatellite markers and several SNPs found through ddRAD-Seq to detect genetic differentiation among populations at continental [16] and city spatial scales [17, 18] using individual *Ae. aegypti*. They found that the SNP loci could detect more distinct genetic differentiation among populations than the microsatellite markers. An additional advantage of ddRAD-Seq over microsatellite markers is the generation of neutral and non-neutral loci that can be used for studying population structure and adaptive divergence simultaneously. However, one limitation of ddRAD-Seq is its high cost, which may preclude the analysis of a large number of individuals. Nevertheless, larger sample sizes in a population are the better option for accurately estimating allele frequencies in the population [19]. To meet these challenges, Pool-Seq, a sequencing strategy that greatly reduces the cost and time of ddRAD-Seq by pooling multiple individual samples, has been developed [20]. Pool-Seq can estimate the gene frequencies of many populations relatively inexpensively because many individuals are available per population. To date, no study has used the Pool-Seq ddRAD approach for analyzing the adaptive divergence of *Ae. aegypti* mosquitoes and comparing the population genetic structure of *Ae. aegypti* mosquitoes with microsatellite markers in the relatively fine-scale area.

Population genetics studies on mosquitoes have mainly focused on female mosquitoes (e.g. *Anopheles gambiae* [21], *Anopheles minimus* [22], *Anopheles arabiensis* [23]; *Aedes albopictus* [24, 25], *Ae. aegypti* [26]), which transmit diseases. However, the population structure of male *Ae. aegypti* populations must also be explored in the context of the function of male mosquitoes in mosquito control strategies, such as, for example, the *Wolbachia*–*Aedes* suppression strategy and the release of sterile male mosquitoes into populations [27]. In Metropolitan Manila, Philippines, Carvajal et al. [28] separated female and male populations using microsatellite markers, which revealed their different dispersal patterns. At the fine-spatial scale, females and males in the same population tend to be highly genetically similar and difficult to separate. Therefore, determining the population structures of female and male *Ae. aegypti* at a fine-spatial scale with confidence requires many neutral markers, such as neutral SNP markers. However, the population genomics of female and male *Ae. aegypti* populations, including their adaptive divergence and population structure on a fine-spatial scale, has not been studied.

The aim of the present study was to determine the population genomic structure of *Ae. aegypti* mosquitoes at a regional spatial scale, namely in Metropolitan Manila. The specific objectives of the study were: (i) to identify the adaptive divergence of *Ae. aegypti* along environmental gradients of climatic and/or landscape factors across the regional scale; (ii) to compare the population divergence levels of female and male *Ae. aegypti*; and (iii) to determine whether a number of SNP loci detected via pool ddRAD-Seq or microsatellite markers are more capable of detecting genetic differentiation among populations on a regional scale. Regarding these aims, we successfully used pool ddRAD-Seq to detect adaptive divergence among *Ae. aegypti* populations along environmental gradients at a relatively fine-spatial scale (< 50 km) in Metropolitan Manila, and we determined dispersal patterns among local populations as well as their sex differences.

## Methods

### Study area

We performed ddRAD-Seq analysis using DNA sampled from 217 *Ae. aegypti* mosquitoes collected from Metropolitan Manila that had previously been used in two population genetic studies involving 11 microsatellite markers [29, 30]. Of these 217 mosquitoes, 165 were collected from 82 households distributed across Metropolitan Manila [29], and 52 were collected intensively from 39 households distributed within a small area (0.048 km^2^) in Manila City, Metropolitan Manila [30]. The samples were collected using a UV light trap (MosquitoTrap; Jocanima Corporation, Las Pinas City, Philippines). The individual insects were identified at species level using the pictorial keys of Rueda et al. [31].

**Fig. 1:**
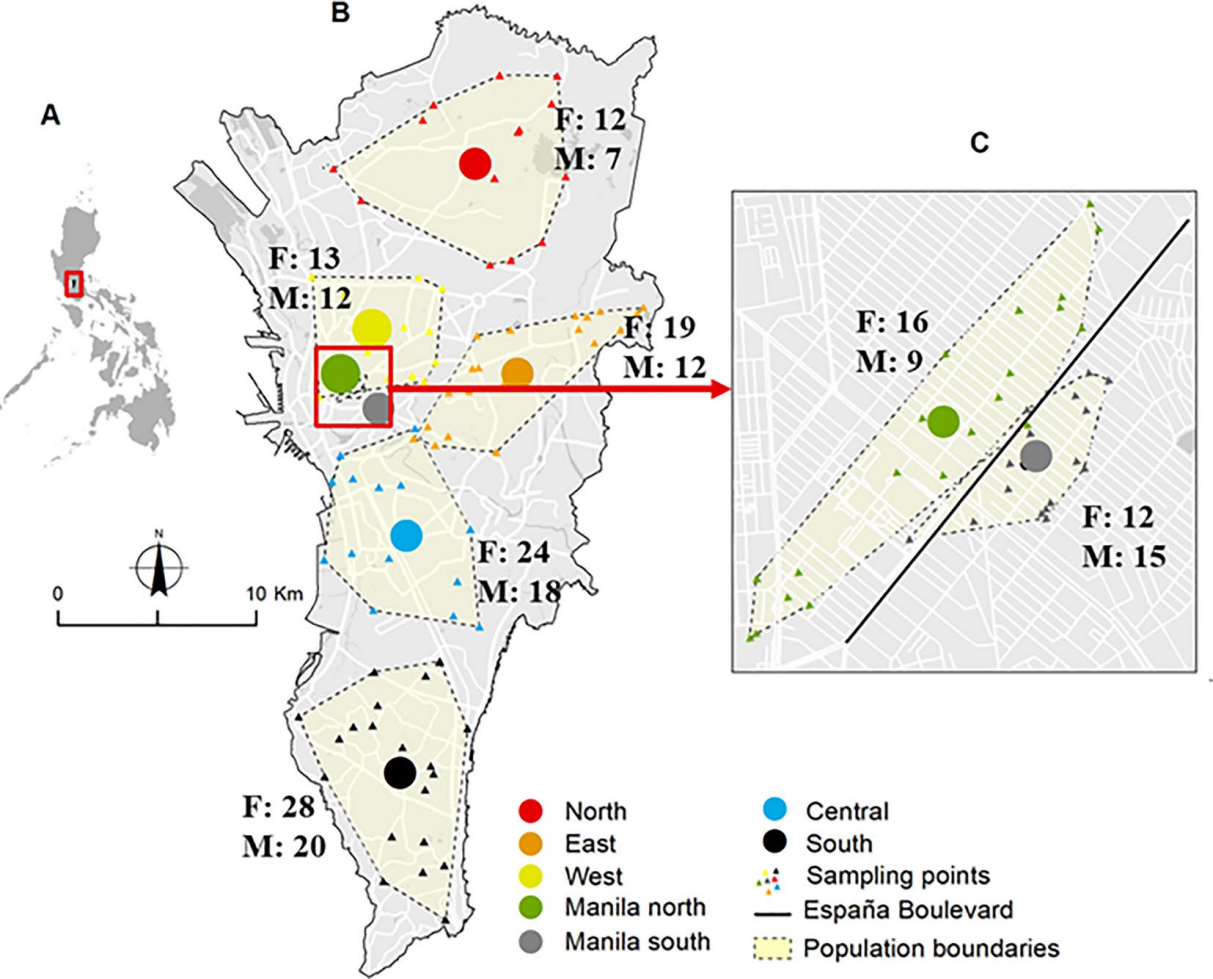
Geographic locations of *Aedes aegypti* collection sites in Manila City (**C**), Metropolitan Manila (**B)** and the Philippines (**A**). Circles indicate the geographical midpoints of *Ae. aegypti* populations per location; triangles indicate the households in the sampling locations. F, Total number of female mosquitoes per population; M, total number of male mosquitoes per population

In this study, we identified 14 populations (7 regions for each gender) by merging neighboring administrative areas such as cities and using the year of sampling. The aim was to ensure a minimum of 10 individuals per population. Despite two populations containing only seven and nine individuals respectively (Fig. 1), we included all 14 populations in our analysis. The number of individuals per male and female populations ranged from 7 to 20 and 12 to 28, with mean values of 13.2 and 17.7 individuals, respectively (Table 1). Of the seven regions, five are subdivisions within Metropolitan Manila, while the other two are smaller regions within Manila City. The five subdivisions in Metropolitan Manila that were studied by Carvajal et al. [28] include North (Quezon City, northern part of Caloocan City and Valenzuela City), West (southern part of Caloocan City, Manila City and Quezon City), East (Marikina City, Pasig City, Quezon City, San Juan City and Mandaluyong City), Central (Pasay City, Manila City, Taguig City and Makati City) and South (Las Pinas City, Paranaque City and Muntinlupa City). The two smaller regions within Manila City, namely Manila North and Manila South, were studied by Regilme et al. [30] (Fig. 1). Carvajal et al. [28] divided Metropolitan Manila into North, South, East, West and Central regions and collected mosquitoes in May 2014 to January 2015. Regilme et al. [30] sampled mosquitoes in September to October 2017 for two populations (North Manila and South Manila) located north and south of the main road, España Boulevard. West of Metropolitan Manila and North Manila and South Manila were defined as separate populations due to the different year of mosquito collection in order to avoid inter-annual variation in mosquito populations affected by seasonal and inter-annual environmental changes (see, for example, [32]). The *Ae. aegypti* samples used in this study were collected from 153 households, with the number of households per male and female mosquito populations ranging from 7 to 15 and 8 to 16, and mean values of 10.1 and 11.7 households, respectively (Table 1). To determine the midpoint of each population, we calculated the geographical midpoint of these households using ArcGIS 10.2 (ESRI, Redlands, CA, USA). Permission was obtained from each household representative prior to the mosquito collection. The total number of male and female *Ae. aegypti* samples was 93 and 124 individuals, with 7–20 and 12–28 individuals per population, respectively (Additional file 1: Table S1).

**Table 1:** Information on *Aedes aegypti* samples from Metropolitan Manila and the associated Watterson's estimator, Tajima's Π and Tajima's* D* values

Region no.	Population	Sex	Number of individuals	Number of households	Watterson's θ	Tajima’s Π	Tajima’s* D*
1	North	Female	12	9	0.012312	0.011551	− 1.05428
		Male	7	7	0.011625	0.010951	− 0.97828
2	East	Female	19	16	0.011152	0.010694	− 0.73386
		Male	12	9	0.01191	0.011321	− 0.8773
3	West	Female	13	10	0.010964	0.010482	− 0.78341
		Male	12	9	0.011839	0.011262	− 0.8428
4	South Manila	Female	12	11	0.010526	0.01011	− 0.69995
		Male	15	8	0.010811	0.010391	− 0.65798
5	North Manila	Female	16	8	0.011994	0.011393	− 0.90801
		Male	9	12	0.011729	0.011184	− 0.83895
6	Central	Female	24	14	0.012583	0.011478	− 1.3228
		Male	18	11	0.012108	0.011434	− 0.9789
7	South	Female	28	14	0.011315	0.010694	− 0.97128
		Male	20	15	0.011587	0.010992	− 0.84192

### ddRAD-Seq library preparation

Before ddRAD-Seq library preparation, the DNA concentration per mosquito was measured using a Quantus fluorometer (Promega, Madison, WI, USA). We pooled 7–28 individuals (Additional file 1: Table S1) with equimolar DNA concentrations per individual (Pool-Seq) [33] in a population pooling scheme.

The DNA of each population pool was digested using the selected restriction enzymes (*Mlu*CI and* Nla*III) [16] for 3 h at 37 °C. This was followed by an enzyme inactivation step during which the DNA was kept at 65 °C for 20 min and then by a purification step (QiaQuick PCR Purification Kit; Qiagen, Hilden, Germany) to remove further restriction enzyme activity in the samples. The digested DNA was then ligated to the modified Illumina P1 and P2 adapters [16]. Adapter ligation was performed using T4 Ligase buffer containing 0.5 µl of 4 nM/µl P1 Adapter, 0.5 µl of 6 nM/µl P2 Adapter, T4 DNA ligase and H_2_O, at 16 °C for 16 h, after which the remaining ligase enzymes were inactivated by treatment at 65 °C for 20 min. To increase the concentration of the adapter-ligated DNA (library), we amplified the library using PCR with a 10-µl reaction mixture containing 5 µl of Phusion High Fidelity MASTER Mix (New England Biolabs, Ipswich, MA, USA), 2 µl of P1 primer (AAT GAT ACG GCG ACC ACC GAG ATC TAC ACT CTT TCC CTA CAC GAC G) and 2 µl of P2 primer (CAA GCA GAA GAC GGC ATA CGA GAT CGT GAT GTG ACT GGA GTT CAG ACG TGT GC). The PCR cycling conditions were: 98 °C for 30 s; followed by 12 cycles of 98 °C for 10 s, 60 °C for 30 s and 72 °C for 90 s; with a final elongation at 72 °C for 5 min. Seven PCR replicates were pooled and purified using the QiaQuick PCR Purification Kit (Qiagen) to form the final library, and then the final library was checked for quality and quantity using the Bioanalyzer and a KAPA Quantification Kit (Roche Diagnostics, Indianapolis, IN, USA), respectively. The libraries were sequenced using the HiSeq X Ten Sequencing System (paired-end, 2 × 150 bp) (Illumina Inc., San Diego, CA, USA) at the Beijing Genomics Institute, China.

### Data processing

All raw sequence data were deposited in the NCBI with the accession number of BioProject PRJNA954465. The raw sequence data were assessed for sequence read quality using FastQC v0.11.8 [34], and FastQC information was used as a guide for trimming and filtering the raw data. Adapters and barcodes were removed using Trimmomatics v.0.39 [35], and the trimmed and filtered data were mapped to the *Ae. aegypti* reference genome AaegL5.0 (www.vectorbase.org/organisms/aedes-aegypti/liverpool-lvp/AaegL5.0) using the *bwa mem* algorithm of the Burrows–Wheeler Aligner (BWA) [36]. The mapping results from BWA generated a mapping file in the Sequence Alignment Map (SAM) format. We filtered out ambiguously mapped reads with a minimum mapping quality score of < 20. The SAM files were converted and sorted to Binary Alignment Map (BAM) files using SAMtools v.1.9 [37] to sort the sequences to the reference coordinates in a memory-efficient file form. All sorted BAM files of all populations were synchronized to the reference genome in the *mpileup* format using SAMtools. The *mpileup* format file was converted to a *sync* file using Java *mpileup2sync.jar* script on Popoolation2 [38]. We did not separate the female and male populations for the SNPs calling because one of the objectives of this study was to compare the population genetic structure of the female and male populations. Detecting different SNP loci in female and male populations and estimating the population genetic structure (e.g., non-metric multidimensional scaling [NMDS]) based on these different SNP loci members, as opposed to using SNP loci members common to both sexes, would complicate a pure comparison of the genetic structures. SNPs were identified and allele frequencies estimated using *snp-frequency-diff.pl* script from Popoolation2 with a coverage range of 15–200 and minimum allele count of 4. The minimum coverage of 15 was selected based on the mean number of samples per pool, simulating a set number of individuals per pool. Popoolation2 was used to perform SNP calls to reference genomes as reference call SNPs (rc SNPs) and SNPs observed between populations (pop SNPs), respectively. The rc SNPs were deleted, whereas the pop SNPs were retained for subsequent analysis. The selected SNP loci were not filtered for Hardy–Weinberg equilibrium (HWE) because we needed to analyze loci potentially under natural selection, which may not theoretically align with HWE for studies of adaptive divergence. Moreover, removing markers not in HWE from the dataset may have only minor or no impact on the estimation of population structure [39].

### Detection of non-neutral SNPs

Non-neutral SNPs were selected using three methods: empirical-, principal component analysis (PCA)- and Bayesian-based methods. In the empirical-based method, we extracted non-neutral SNPs in the lower and upper 1% tails of a frequency distribution of pairwise fixation index (*F*_ST_; a measure of population differentiation due to genetic structure values), as estimated via Popoolation2 using the *fst-sliding.pl* script. SNPs detected in the lower tail were considered to be balancing selection candidates, whereas SNPs detected in the upper tail were considered to be divergent selection candidates [40]. The pcadapt v.4.3.3 package in RStudio was employed to analyze Pool-Seq data [41]. In this analysis, the Benjamini–Hochberg procedure was used to decrease the false discovery rate with alpha value 0.05 (expected false discovery rate < 5%) during non-neutral SNPs detection [42]. The Bayesian-based method was employed using BayeScan 2.1 [43]. The input file used for BayeScan v.2.1 was in the *bayenv* file format; therefore, we created a GenePop file from the *sync* file using the *subsample_sync2GenePop.pl* script in Popoolation2. The GenePop file was then edited and converted into the *bayenv* format using PGDSpider v.2.1.1.5 [44]. BayeScan was run with 20 pilot runs, an additional burn in value of 50,000 and a thinning interval of 10 with sample size 5000. To further reduce false positive non-neutral SNP detection, we defined non-neutral SNPs as those detected by all three methods. Subsequently, the detected non-neutral SNPs were removed from the complete dataset, and only the neutral SNPs dataset was retained for population structure analysis.

### Genetic diversity and population genetic structure

Genetic diversity per population was calculated using NPStat v.1.0 [45] by estimating the population mutation rate (Watterson’s estimator theta [*ϴ*]) and nucleotide diversity (Tajima’s phi [*π*]) from a *pileup* file using a minimum Phred score of 20, coverage range of 15–200 and minor allele count of 4. Population genetic structure was analyzed using mean pairwise *F*_ST_ values across neutral SNPs via NMDS and permutational multivariate analysis of variance (PERMANOVA) analyses in the R package vegan v. 2.5–7 [46]. Mean pairwise *F*_ST_ values from neutral SNPs were also used to test isolation by distance for female and male populations by conducting the Mantel test via GenAlex v.6.5 [47] and using a geographical distance matrix (km). Geographical distance was calculated based on the geographical midpoints of each population. The neutral SNPs from chromosome 1 were removed from the population genetic structure analysis to avoid the potential bias generated by sex-linked markers located in this chromosome. The global *F*_ST_ values of female and male populations were calculated and then tested separately for neutral and non-neutral SNPs using the Wilcoxon rank-sum test in RStudio.

### Microsatellite analyses and comparison of microsatellite markers with neutral SNPs

To enable a comparison with the neutral SNP data obtained in the present study, we calculated the pairwise *F*_ST_ values among the 14 populations using previously obtained genotype data of 11 microsatellite markers at a regional scale [29] and at a smaller spatial scale in Manila [30]. However, because different capillary electrophoresis instruments were used for fragment analysis in the previous studies [29, 30], we separated the comparative analysis of 10 [29] and four [30] populations, respectively, to avoid bias due to instrumental differences. Microsatellite data were analyzed using Arlequin v. 3.5 [48] to calculate pairwise *F*_ST_ values. The unweighted pair-group method with arithmetic (UPGMA) dendrograms from the microsatellites and neutral SNPs of the 10 populations [29] were generated using Phylip-3.698 [49]. Additionally, a Mantel test was performed via GenAlex v.6.5 [47] to test the correlation of pairwise *F*_ST_ values between microsatellite and neutral SNP markers in these 10 populations [29].

### Environmental association and gene annotation analyses of non-neutral SNPs

The non-neutral SNPs were used in the environmental association analysis in which the environmental variables consisted of five climatic and eight landscape variables. We used the mean values of climatic variables (i.e. precipitation, air temperature, relative humidity, northward wind and eastward wind) per population according to satellite-based remote sensing data obtained from the Google Earth Engine code editor platform [50] with an identical data duration and sampling collection time (Additional file 1: Table S2). Additional preprocessing to fill missing pixel data was performed using GRASS GIS software version 7.8.3 (GRASS Development Team, https://grass.osgeo.org/). We used landscape data published by Francisco et al. [51], which included the percentage of the area of the following landscape categories in each village in 2014–2015 and 2017: water bodies, grassland, agricultural, open spaces, parks and recreational areas, residential areas, forests and buildings (education, health, and welfare; religious and cemetery; military; governmental institutions; industrial; commercial; transport; and informal settlements) (Additional file 1: Tables S3, S4, S5).

To assess the association between changes in allele frequencies in non-neutral SNPs and environmental conditions, we conducted distance-based redundancy analysis (db-RDA) using the *capscale* function and the variable selection algorithm via the *ordistep* function in the R package vegan v. 2.6–4 [46]. We used all climatic and landscape variables as explanatory variables and the pairwise genetic differences (*F*_ST_) matrix of each non-neutral locus as a response variable. To understand the respective adaptive responses of males and females to environmental variables, we conducted the db-RDA of male and female populations separately.

The non-neutral SNPs were annotated for candidate genes using blastx from the NCBI with its default parameters [52]. We used the position of the non-neutral SNPs in the chromosome to blast it in blastx toward the *Ae. aegypti* reference genome (AagL5.0) (https://www.ncbi.nlm.nih.gov/datasets/genome/GCF_002204515.2/) to determine if they exhibited functional response. The biological functions of the identified genes were investigated based on 259 categories of Gene Ontology annotations in the Universal Protein Knowledgebase (www.uniprot.org).

## Results

### ddRAD-Seq and non-neutral SNPs detection

In total, 377,047,648 raw reads with a length of 150 bp were generated via ddRAD-Seq, with 18,059,405–40,849,752 reads obtained per population. After trimming the adapters and filtering the low-quality reads, 38,181,713 reads were removed and 338,865,935 reads were retained. We identified 65,473 SNPs, with 1880 and 2401 SNPs found exclusively among female and male populations, respectively.

**Fig. 2:**
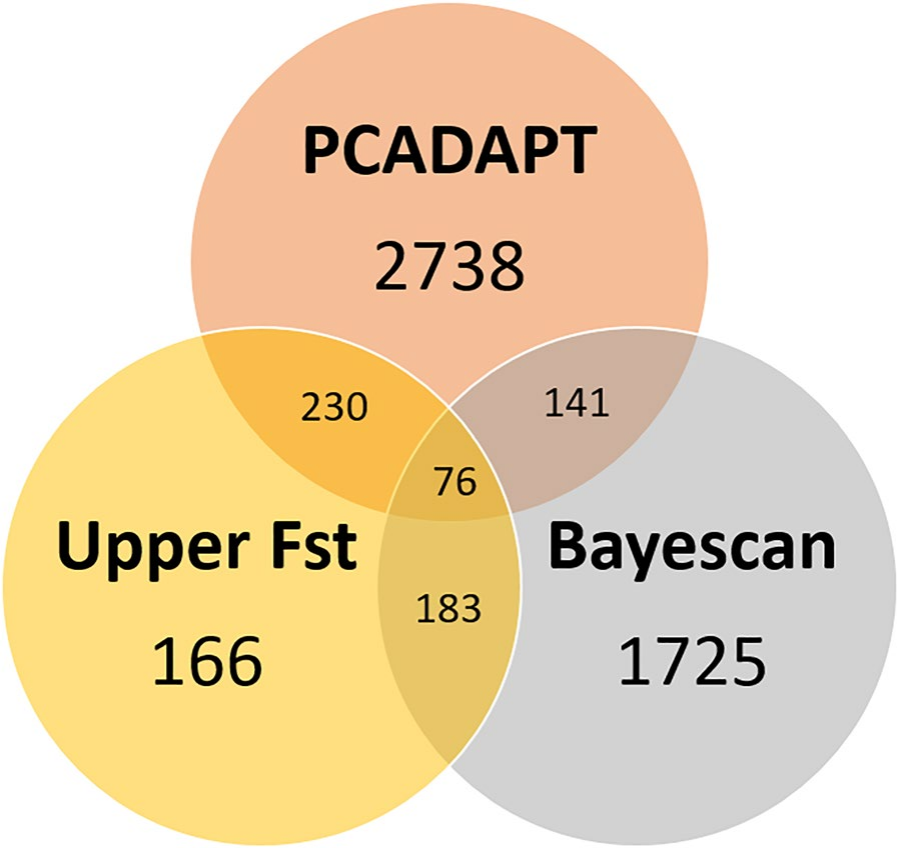
The numbers of non-neutral SNPs detected from 65,473 SNPs, using three different methods: *F*_ST_ distribution, PCA-based approach (PCADAPT) and Bayesian-based approach (Bayescan). The 76 SNPs detected by these three methods were considered to be non-neutral SNPs in subsequent analyses. *F*_ST_, Pairwise fixation index; PCA, principal component analysis; SNP, single nucleotide polymorphism

The empirical-based method, PCADAPT, and BayeScan detected 655, 3185 and 2125 non-neutral SNPs, respectively. Overall, 76 non-neutral SNPs were detected by all three non-neutral detection methods (Fig. 2; Additional file 1: Figures S1, S2). In subsequent analyses, the 76 non-neutral SNPs detected by these three non-neutral/outlier detection methods were classified as non-neutral SNPs, whereas the other SNPs were classified as neutral SNPs. blastx searches found that 49 non-neutral SNPs were located on or close to genes associated with enzymatic activity, nucleic acid-binding and metabolism activity (Additional file 1: Table S6).

### Genetic diversity and population genetic structure

**Fig. 3:**
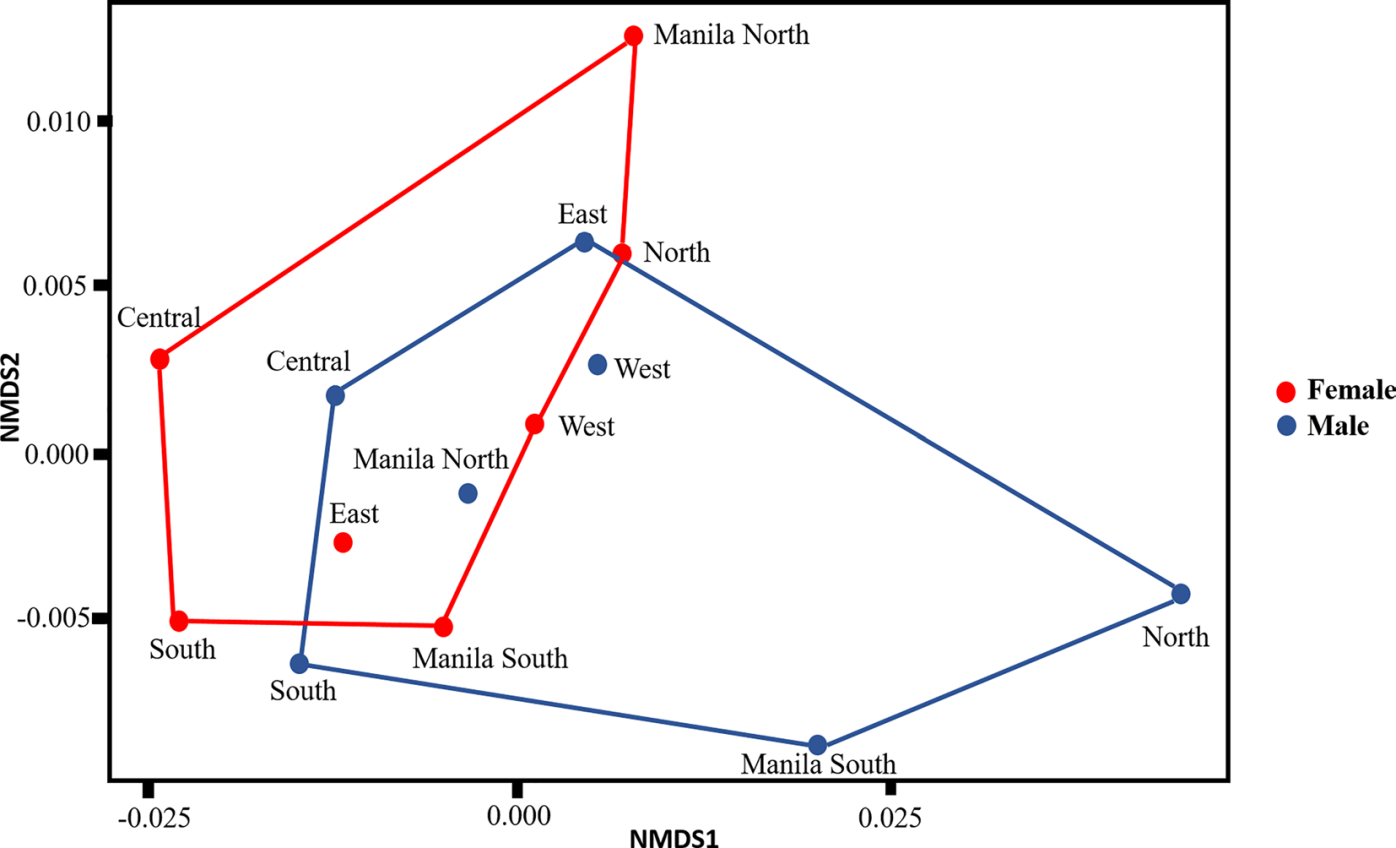
Population genetic structure of male (blue) and female (red) *Aedes aegypti* populations according to non-metric multidimensional scaling (NMDS) based on neutral SNPs without chromosome 1. PERMANOVA analysis revealed no significant divergence between the female and male populations (*R*^2^ = 0.09344, *p* = 0.225). PERMANOVA, Permutational multivariate analysis of variance; SNP, single nucleotide polymorphism

Watterson’s *ϴ* and Tajima’s *π* of neutral and non-neutral SNPs indicated low genetic diversity throughout all populations (Watterson’s *ϴ* = 0.011–0.012; Tajima’s *π *= 0.010–0.011) (Table 1). Mean pairwise *F*_ST_ values among the populations were 0.022–0.069 (overall mean =  0.038) for neutral SNPs and 0.042–0.23 (overall mean = 0.120) for non-neutral SNPs. Mean global *F*_ST_ values were compared across neutral SNPs using a Wilcoxon rank-sum test; significant differences were found between female and male populations (*P* < 0.0001), but no significant differences were found across non-neutral SNPs (*P* = 0.071). In addition, there was no isolation by distance in both female (*R*^2^ = 0.0871*, P* = 0.091) and male populations (*R*^2^ = 0.28*, P* = 0.095) (Additional file 1: Figure S3). PERMANOVA of the neutral SNPs indicated that no significant difference existed between female and male populations (*R*^2^ = 0.09344, *P* = 0.2244), and NMDS plots revealed an unclear separation between the female and male populations based on neutral SNPs without the SNPs from chromosome 1 (Fig. 3). However, as shown in Fig. 3, we still observed the separation between the female and male populations. The populations from West, East, Manila South Female and Manila North Male appeared to overlap in the NMDS analysis result; this might due to the close distance between the populations (Fig. 3), and they might be genetically closed. Male populations exhibited a more diverse structure than female populations, with the North and South Manila populations being genetically isolated from the other populations.

### Comparison of neutral SNP and microsatellite markers

**Fig. 4:**
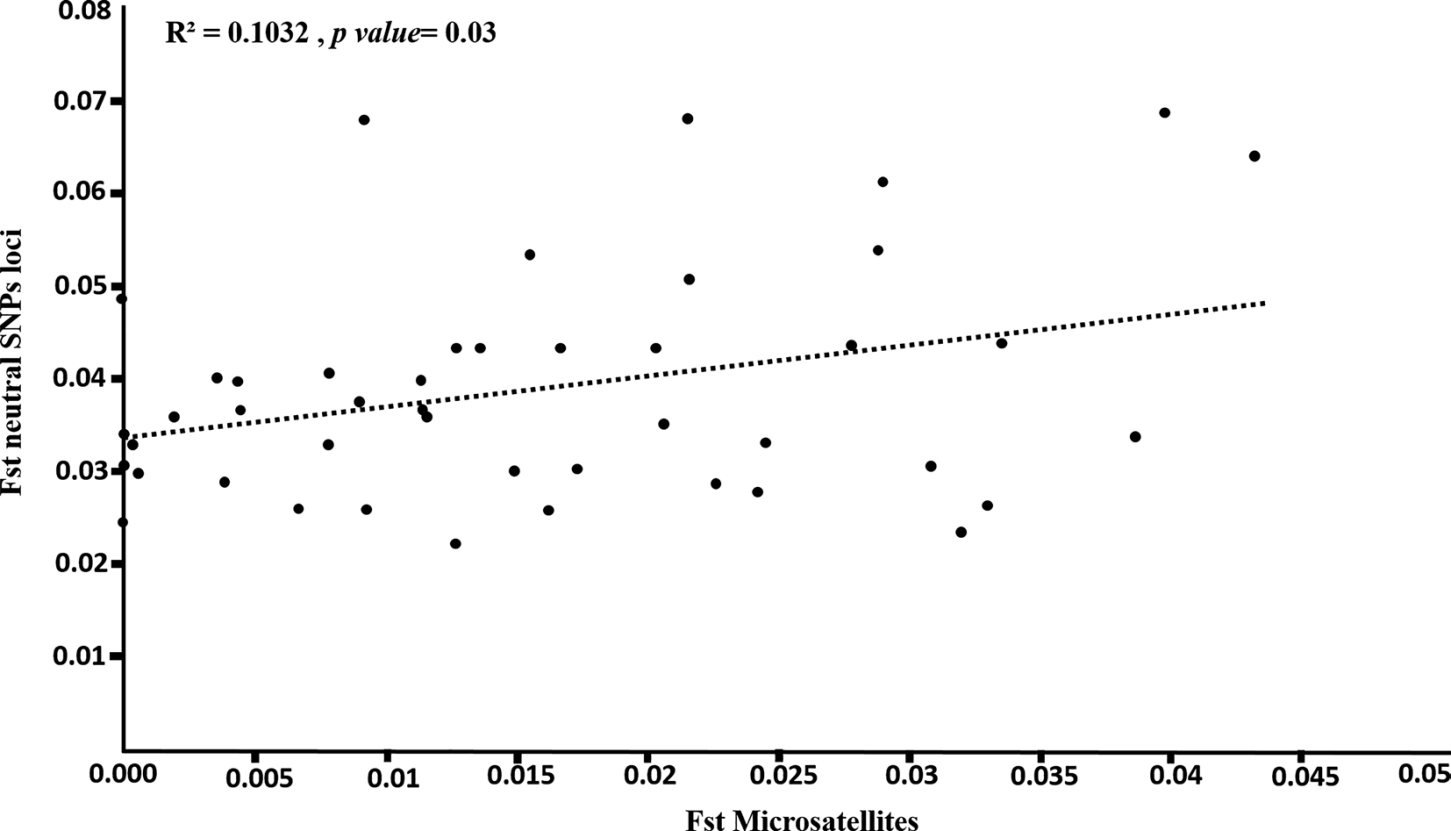
Regression of pairwise genetic differentiation (*F*_ST_) of neutral SNP and microsatellite markers obtained using data from Metropolitan Manila. *F*_ST_, Pairwise fixation index; SNP, single nucleotide polymorphism

In the 10 studied populations in Metropolitan Manila, the pairwise *F*_ST_ values generated from neutral SNPs (0.022–0.069; mean = 0.038) were higher than those generated from microsatellite markers (0–0.043; mean = 0.015). According to the Mantel test, the pairwise *F*_ST_ values obtained from neutral SNPs and microsatellite markers were significantly correlated (*R*^2^ = 0.1032, *P* = 0.03; Fig. 4) with a regression equation of *y* = 0.3327x + 0.0336.

**Fig. 5:**
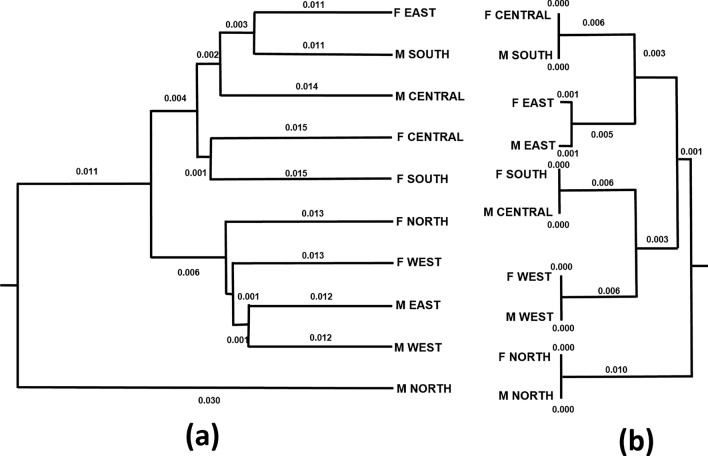
“Unweighted pair group method with arithmetic mean” dendrograms based on mean pairwise genetic differences (*F*_ST_) among 10 regional-scale populations in Metropolitan Manila according to neutral SNP loci (**a**) and microsatellite markers (**b**). F, Female;* F*_ST_, pairwise fixation index; M, male; SNP, single nucleotide polymorphism

Figure 5 shows a comparison of the dendrograms of the 10 *Ae. aegypti* populations in Metropolitan Manila constructed using the pairwise *F*_ST_ values of microsatellite and neutral SNP markers. The dendrogram generated from microsatellite markers had shallow branch lengths (0.010) from the terminals to the common ancestor. In contrast, in the dendrogram generated from neutral SNPs, the overall branch length from the terminal to the common ancestor was longer (0.030), indicating the unique genetic structure of individual populations. Additionally, based on the dendrogram of microsatellites markers (Fig. 5b), eight populations (Female North with Male North; Female West with Male West; Female East with Male East; and Female South with Male Central) were not separated in the dendrogram. In addition, based on the use of neutral SNPs, all pairs of populations were separated from each other (Fig. 5a). At a smaller scale, i.e. within Manila, the *F*_ST_ values of the neutral SNP of Female North–South Manila and Male North–South Manila were slightly higher (0.037 and 0.040, respectively) than those of microsatellite markers (0.037 and 0.024, respectively).

### Association of non-neutral SNPs and environmental variables

**Fig. 6:**
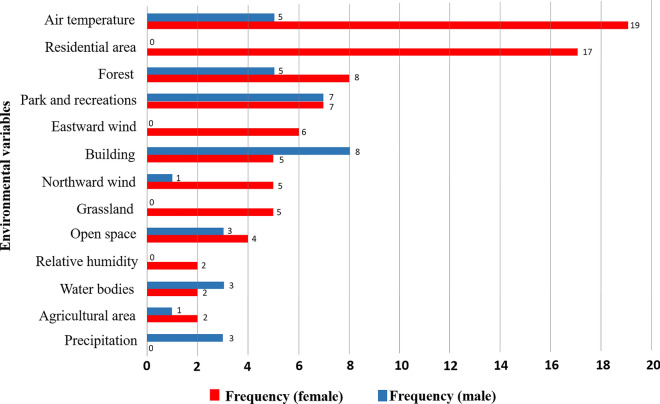
Frequency of 50 and 29 non-neutral SNPs from female and male populations, respectively, associated with environmental variables selected in distance-based redundancy analysis models. SNP, Single nucleotide polymorphism

There is variation of environmental variables across metropolitan Manila, Philippines (Additional file 1: Table S3). db-RDA analysis revealed that of the 76 non-neutral SNPs, 29 from male populations and 50 from female populations were significantly associated with 13 environmental variables. Of these non-neutral SNPs, 20, four and five were associated with landscape variables, climatic variables, and both landscape and climatic variables, respectively, in the male populations, whereas 21, 10 and 19 non-neutral SNPs were associated with landscape variables, climatic variables and both landscape and climatic variables in the female populations. Air temperature was associated with the highest number of non-neutral SNPs (*n* = 19), followed by residential area (*n* = 17), forest (*n* = 8) and parks and recreational area (*n* = 7) in the female populations. In the male populations, parks and recreational area together with buildings were associated with the highest number of non-neutral SNPs (*n* = 8), followed by park and recreation (*n* = 7), forest (*n* = 5) and air temperature (*n* = 5) (Fig. 6).

## Discussion

### Adaptive divergence of *Ae. aegypti* populations

In the present study, 76 non-neutral SNPs were detected in *Ae. aegypti* populations at a fine scale, i.e. in Metropolitan Manila; of these, 26 SNPs were associated with landscape features and 10 with climate in male populations whereas 40 SNPs were associated with landscape features and 26 with climate in female populations (Fig. 6). This result may have been due to the spatially homogeneous climatic conditions across Metropolitan Manila (Additional file 1: Table S3), despite there is genetic divergence among the 14 studied populations. The measurement of the spatial heterogeneity of microclimates at an intra-urban scale, such as in residential landscapes, is challenging, as microclimates are homogenous [53, 54].

In female populations, of the 50 non-neutral SNPs associated with both landscape and climatic variables, 19 were associated with air temperature, 17 were associated with residential area, eight were associated with forest and seven were associated with parks and recreational area (Fig. 6). These results are consistent with those of Bennett et al. [14] which showed that the genetic variation of SNPs was correlated with temperature and vegetation. They are also consistent with the results of a another study [13] which revealed that seven loci were associated with human density. In male populations, eight non-neutral SNPs were associated with buildings, seven were associated with parks and recreational area and five were associated with forest and air temperature (Fig. 6). *Aedes aegypti* mosquitoes are known to exhibit endophilic behavior (i.e. taking shelter inside the house) and endophagic behavior (i.e. blood-feeding inside houses). They have also been observed to move from inside the house to outside the house (or vice versa) [55]. Female *Ae. aegypti* are known to exhibit long-distance movement facilitated by humans [56, 57], and it is likely that they experience seasonal and daily fluctuations in temperature from one to another place. Air temperature also affects the flight patterns of female *Ae. aegypti* mosquitoes. They tend to fly for short periods of time in the temperature range between 10 °C and 35 °C [58]. Seventeen non-neutral SNPs in female populations were associated with residential area. This may relate to the blood-feeding behavior of the female *Ae. aegypti* mosquito, which has anthropophagic behavior (preferring humans for blood-feeding) [59–61]. Both male and female populations showed a high association of non-neutral SNPs with vegetation-related areas, such as parks, recreational areas and forests. These areas are known to be crucial for reproduction- and survival-related fitness in *Ae. aegypti,* and they are abundant in urban green spaces [62, 63]. Additionally, both male and female *Ae. aegypti* mosquitoes rely on vegetation as a source of sugar and as resting places to aid blood ingestion [64]. Vegetation also supplies local moisture, which supports the activity and survival rate of mosquitoes [65, 66]. Medically important mosquitoes, such as *Ae. aegypti* and *Ae. albopictus*, are found in parks and green spaces in urban areas [67, 68]. According to evolutionary theory, fitness and adaptive divergence are related [69, 70], i.e. high fitness or suitability to a specific environmental condition may increase the local density of a species, leading to high intraspecific competition for space or resources. Consequently, some populations adaptively evolve to alternative environmental conditions to avoid competition, leading to adaptive divergence along environmental gradients [71]. Although we did not measure mosquito abundance or the level of intraspecific competitive pressure in the present study, we found that environmental factors potentially cause adaptive divergence at many non-neutral SNPs, which is associated with mosquito fitness and supports the aforementioned theory.

In this study, the non-neutral SNPs were located on or near the genes and/or proteins related to nucleic acid binding, metal ion binding, ATP binding, DNA biosynthetic process and catalytic activity. One of the non-neutral SNPs identified in this study is associated with the lava lamp protein isoform X1. The lava lamp protein is *Drosophila* golgin protein that is essential for the early stage of *Drosophila* embryogenesis [72]. Embryogenesis is the first and most important stage of the insect life-cycle. Early physical environmental effects may influence embryo development since embryo development of insect occurs outside the mother’s body [73]. Non-neutral SNPs in chromosome 3 (position: 295070690) were found to be located in the stress response protein NST1-like. NST1 protein is involved in the heat stress response through the cell wall integrity (CWI) pathway [74]. In the same chromosome 3 (position: 309404428), fatty acyl-CoA reductase 1 has been reported to be involved in the biosynthesis of *Apis mellifera* pheromones [75] that are related to insect reproduction. Environmental conditions influence the pheromone signal transmission [76]. The identification of non-neutral SNPs linked to these functions may therefore suggest the possible selective pressures affecting different physiological functions in *Ae. aegypti* related to landscape/climate adaptation.

### Contrasting spatial genetic structures of male and female *Ae. aegypti* populations and comparison between SNPs with microsatellite markers

The spatial population genetic structures of female and male *Ae. aegypti* exhibited contrasting patterns according to neutral SNPs, i.e. male populations exhibited greater spatial divergence than female populations (Fig. 3). Medeiros et al. [77] previously found that the dispersal ability of male and female mosquitoes was different. This pattern is also consistent with that reported in another study in Metropolitan Manila [28] in which wing geometry and microsatellite markers were employed as markers. The different dispersal abilities of male and female mosquitoes might be due to differences in their behavior, such as sex-specific feeding preference and host-seeking behavior [28, 78]. In addition, higher genetic variation in male populations suggests gene flow may be low between male populations, reflecting their low dispersal ability [78]. Maciel-De-Freitas et al. [79] investigated the dispersal and survival rates of *Ae. aegypti* in Rio de Janeiro and found that female mosquitoes move farther than males (mean flight distance: females = 40.94–78.81 m; males = 32.02–42.26 m) and tend to live longer than males. Female *Ae. aegypti* mosquitoes can fly long distances in search of blood meals or oviposition sites when these resources are not available within close range. Indeed, gravid female *Ae. aegypti* can fly up to 3 km to find a suitable egg-laying site [78]. Female *Ae. aegypti* also interact with humans more than their male counterparts, which provides females with more opportunity to experience long-distance passive dispersion via human transportation networks [28]; this would also contribute to the increased flight distance of female mosquitoes during host-seeking behavior. However, in this explanation of the different dispersal abilities of male and female mosquitoes, the different number of SNPs found in each sex is not considered. Indeed, both sex differences in dispersal ability and the higher number of polymorphic loci in the male genome may have contributed to the high spatial genetic divergence detected in males.

We found a significant positive correlation between the pairwise *F*_ST_ values of neutral SNPs and microsatellite markers (Fig. 4), which is consistent with previous studies on Atlantic salmon, *Arabidopsis halleri*, Gunnison sage-grouse, corn rootworm and alpine-endemic birds [80–85]. In addition, we found that the pairwise *F*_ST_ values of neutral SNP markers were higher than those of microsatellite markers, indicating higher resolution in terms of measuring population differentiation. In Fig. 4, the intercept of the regression line (0.0336) indicates that neutral SNP markers can detect a certain amount of genetic differentiation (i.e. pairwise *F*_ST_ = 0.0336), even among populations in which microsatellite markers do not detect any genetic differentiation.

Genetic variation determined at a small spatial scale, such as in Metropolitan Manila, tends to be low (e.g. isolation by distance [86]). However, pool ddRAD-Seq can detect a large number of SNPs, thereby increasing the sensitivity for the detection of low genetic variation that cannot be detected with microsatellite markers. Previous studies [87] revealed that, at a fine-spatial scale, microsatellite markers perform better than a small number of SNP markers for determining population differences because of their high mutation rate. Although microsatellite markers are highly polymorphic, only a small number are usually used owing to logistical constraints [88]. In contrast, Ryynänen et al. [80] found that a small number of SNP markers and microsatellite markers provided comparable results. At a fine-spatial scale, the genetic differences among individuals in different populations are difficult to distinguish because they can share high kinship owing to their adjacent habitat. To determine genetic variation between populations on a small spatial scale, a high number of SNP loci should be used to detect population structure [80, 81]. A few thousand SNPs identified via NGS, compared with several microsatellite markers, are sufficient to estimate the genetic diversity and divergence in natural populations [81, 82].

We applied the Pool-Seq approach with ddRAD-Seq. In recent years, this approach has increasingly been used for population genomic studies [89–94]. When taking an individual-based approach, only a limited number of individuals are selected for analysis from a population owing to cost constraints, and sampling errors that occur when selecting these individuals can lead to erroneous allele frequency estimates in the population. In contrast, in Pool-Seq, libraries are constructed per population rather than per individual, which markedly reduces the resources (e.g. reagents) and time required to complete the analysis. Thus, many more individuals per population can be analyzed, reducing the sampling error and increasing the accuracy of allele frequency estimation [33]. Notably, the Pool-Seq approach has several limitations. First, it cannot recognize the haplotypes of each individual; thus, some analyses, such as STRUCTURE [95] (a widely used individual-based population genetic analysis method), cannot be performed with Pool-Seq. Second, if the amount of template DNA for each individual is unequal when pooled samples are prepared, heterogeneity increases substantially during PCR amplification and may reduce the accuracy of allele frequency estimation [33]. Therefore, in the present study, in each population pool, we mixed the same amount of DNA per individual in a population. Since we collected *Ae. aegypti* samples from spatially distant households within populations, the mean values of the environmental variables for the populations may not accurately represent the environmental characteristics of each population. Additionally, we estimated allele frequencies by pooling individuals from multiple sites or same sites that might be belong to same population or different population with same or different local environmental conditions and assessed the adaptive variance among populations based on the mean values of environmental variables in each population. We acknowledge that these limitations may have reduced the accuracy of our tests for adaptive divergence among populations. Furthermore, a pool of several individuals located several kilometers apart or at the same location may induce the Wahlund effect (i.e. reduction of heterozygosity), which can reduce heterozygosity within populations through the possible population division occurring in the same or relatively far sampling sites. Also, in our study, the sample sizes varied across populations, with the smallest sample consisting of seven individuals. This small sample size may have affected the estimation of allele frequencies. Nevertheless, the creation of a large number of SNP markers in this study statistically mitigates potential biases in allele frequency estimation. It is well-documented that a substantial number of SNP markers can effectively assess population genetic structure, even in cases where the sample size per population is small [96–98]. Additionally, given that most populations had ample sample sizes (> 12 individuals, with an average of 15.5), the influence of small sample sizes on the overall estimated population genetic structure and the association between non-neutral loci and environmental variables may have been limited. Nonetheless, future research may benefit from additional efforts to increase sample sizes and collection endeavors per population for a more precise estimation of allele frequencies.

If the Pool-Seq strategy is applied in the future to study population genetic structure, it would be advisable to improve the spatial scale, increase the number of sites and include more individuals in each pooled population. However, this should be balanced with the effort required to collect samples. Additionally, we suggest using an individual-based approach to study adaptive divergence. This will help to minimize potential bias caused by different environmental conditions between sampling locations and provide a more accurate assessment of the adaptive divergence along a specific environmental gradient.

## Conclusions

We used a pool ddRAD-Seq approach to detect adaptive divergence along environmental gradients and dispersal patterns among *Ae. aegypti* populations, as well as their sex differences at a fine-spatial scale in Metropolitan Manila. The analysis of non-neutral SNPs revealed that spatial heterogeneity in landscape factors linked to mosquito fitness may lead to adaptive divergence in the *Ae. aegypti* populations of Metropolitan Manila. Additionally, neutral SNPs generated through pool ddRAD-Seq proved to be more sensitive in detecting genetic differences between populations at fine-spatial scales compared to 11 microsatellite markers. Interestingly, male populations exhibited greater spatial divergence than female populations. Accurate estimation of male and female *Ae. aegypti* mosquito dispersal at a fine-spatial scale holds potential for designing and implementing vector control programs, such as *Wolbachia*-infected mosquito mass-release programs and sterile insect technique (SIT). These programs would benefit from detailed information on male and female mosquito population dispersal patterns and potential barriers to mosquito movement in and around the release area [99].

It is also imperative to consider the adaptability of *Ae. aegypti* populations toward the environment in the dengue endemic area, where a spatial forecast model will be implemented. By incorporating adaptability as a parameter and combining it with the environmental response, future estimates can be more accurate [14].

### Supplementary Information

**Additional file 1: Figure S1.** Q-Q plot generated using PCA-based detection method. The majority of the *P*-values appear to match the expected uniform distribution, according to this plot. We employed the Benjamini-Hochberg procedure to decrease the false discovery rate with the alpha value of 0.05 (expected false discovery rate < 5%). **Figure S2.** The Bayescan 2.1 plot of the SNPs from this study and detection of the non-neutral SNPs based on the *q*-value threshold FDR (False Discovery Rate) = 0.05. **Figure S3.** Mantel test for detecting isolation by distance between male populations (**a**) and female populations (**b**) using the neutral SNPs dataset. Both male and female populations showed no isolation by distance (*P* >0.05). **Table S1.** Individual Ae.*aegypti* geographical coordinate information. **Table S2.** Characteristics of satellite data obtained from Google Earth Engine. **Table S3.** Environmental variables mean value per sampling region/population. **Table S4.** Correlation matrix between environmental variables, with correlation coefficient shown at top right, and* P*-values at bottom left. **Table S5.** Association between non-synonymous SNPs and environmental variables based on the variable selection analysis. **Table S6.** Within-gene or near-gene SNPs among the putative outlier identified using PCA-, Bayesian-- and Fst-based empirical detection methods.

## Data Availability

The datasets generated in this study were deposited in the NCBI with the accession number of BioProject: PRJNA954465.
