# Supplementary material for: Fine-scale adaptive divergence and population genetic structure of Aedes aegypti in Metropolitan Manila, Philippines
Source: Parasit Vectors. 2024 May 21;17:233. doi: 10.1186/s13071-024-06300-x (PMC11107013; doi:10.1186/s13071-024-06300-x)
Supplement: Supplementary file 1 — Additional file 1: Figure S1. Q-Q plot generated using PCA-based detection method. The majority of the P-values appear to match the expected uniform distribution, according to this plot. We employed the Benjamini-Hochberg procedure to decrease the false discovery rate with the alpha value of 0.05 (expected false discovery rate < 5%). Figure S2. The Bayescan 2.1 plot of the SNPs from this study and detection of the non-neutral SNPs based on the q-value threshold FDR (False Discovery Rate) = 0.05. Figure S3. Mantel test for detecting isolation by distance between male populations (a) and female populations (b) using the neutral SNPs dataset. Both male and female populations showed no isolation by distance (P >0.05). Table S1. Individual Ae.aegypti geographical coordinate information. Table S2. Characteristics of satellite data obtained from Google Earth Engine. Table S3. Environmental variables mean value per sampling region/population. Table S4. Correlation matrix between environmental variables, with correlation coefficient shown at top right, and P-values at bottom left. Table S5. Association between non-synonymous SNPs and environmental variables based on the variable selection analysis. Table S6. Within-gene or near-gene SNPs among the putative outlier identified using PCA-, Bayesian-- and Fst-based empirical detection methods. [file 13071_2024_6300_MOESM1_ESM.docx]

**Supplementary Material**

1. **Supplementary figures**


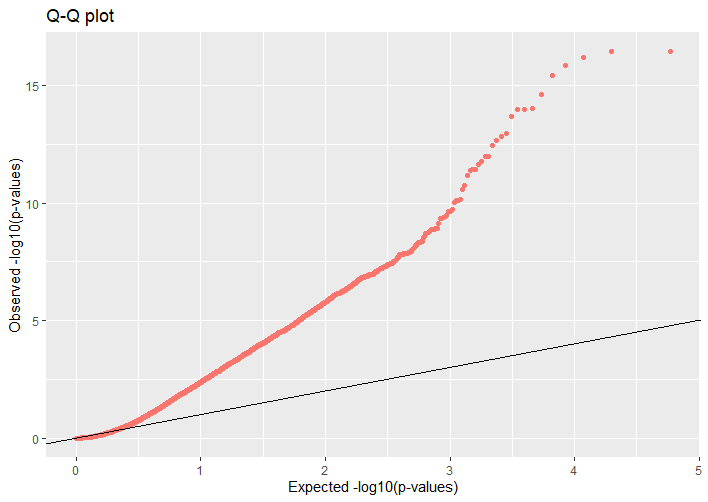


Fig. S1 Q-Q plot generated using PCA-based detection method. The majority of the p-values appear to match the expected uniform distribution, according to this plot. We employed the Benjamini-Hochberg procedure to decrease the false discovery rate with the alpha value 0.05 (expected false discovery rate lower than 5%).


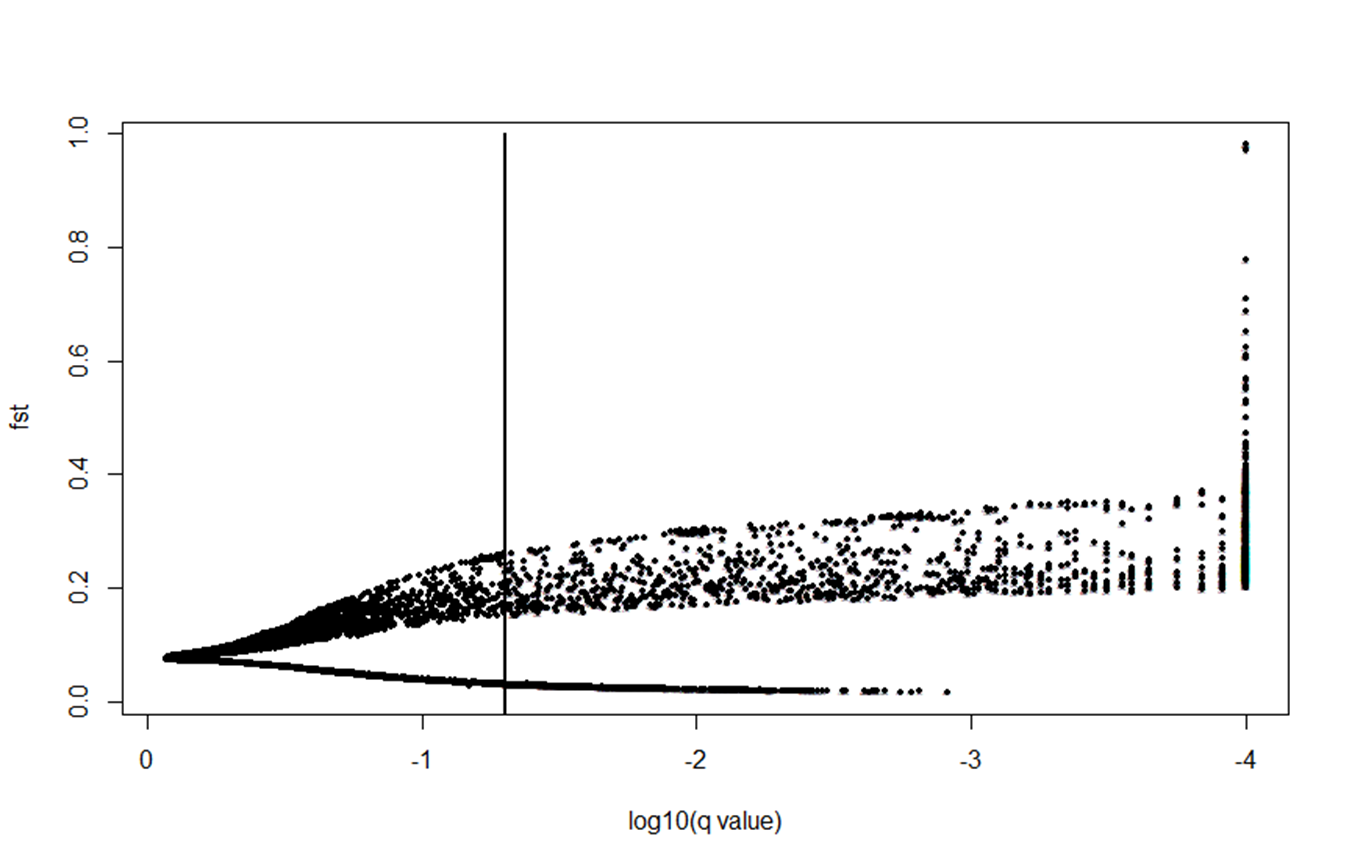


Fig. S2 The Bayescan 2.1 plot of the SNPs from this study and detect the non-neutral SNPs based on the q-value threshold FDR (False Discovery Rate) = 0.05.


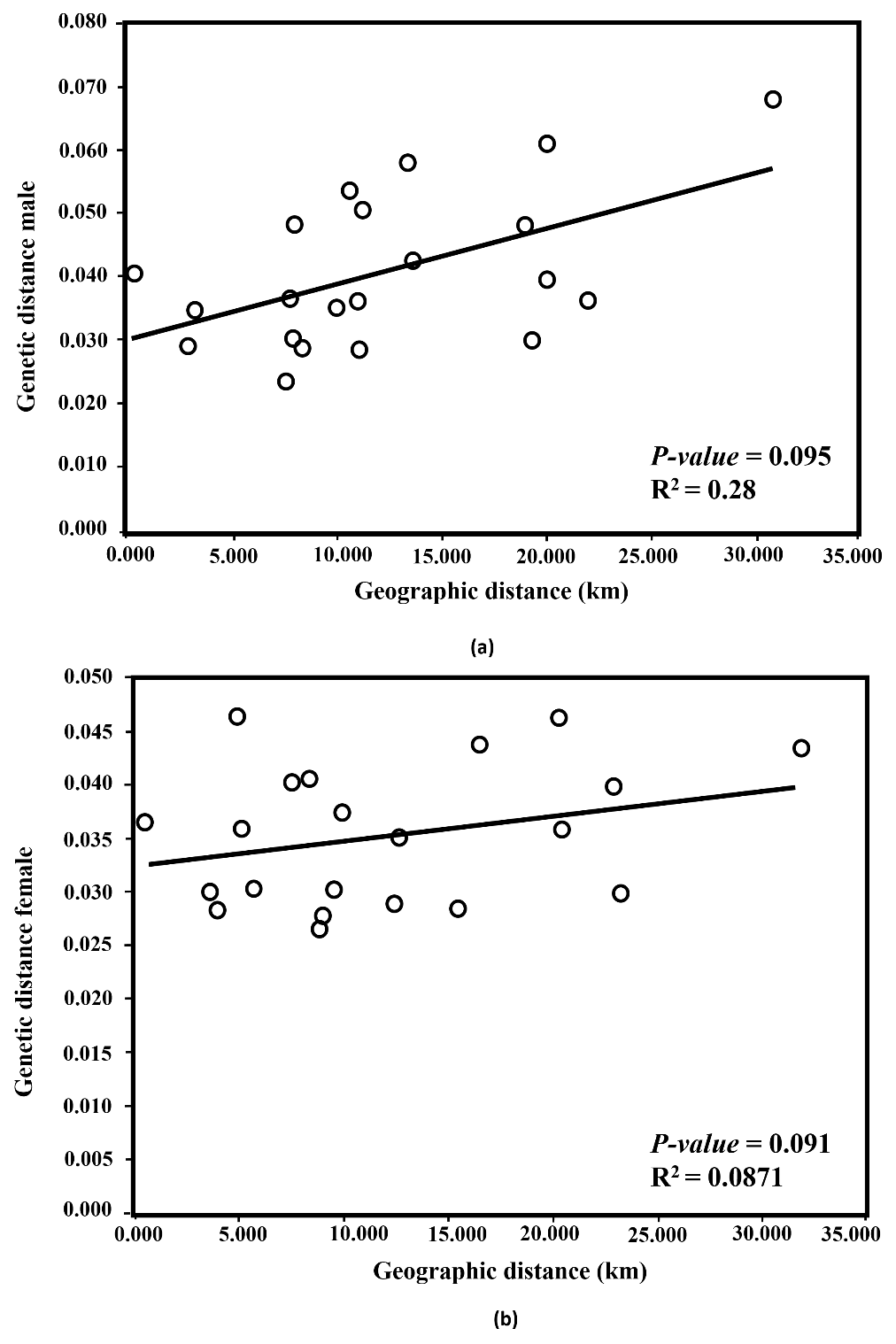


Fig S3. Mantel test for detecting isolation by distance between male populations (a) and female populations (b) using neutral SNPs dataset. Both male and female populations showed no isolation by distance (p>0.05).

1. **Supplementary tables**

**Table S1** Individual *Ae.aegypti* geographical coordinate information

| **No** | **Population** | **Sex** | **Latitude** | **Longitude** |
| --- | --- | --- | --- | --- |
| 1 | North | Female | 14.70972 | 121.0548 |
| 2 | North | Female | 14.70972 | 121.0548 |
| 3 | North | Female | 14.70972 | 121.0548 |
| 4 | North | Female | 14.71156 | 121.088 |
| 5 | North | Female | 14.68173 | 121.078 |
| 6 | North | Female | 14.67304 | 121.0639 |
| 7 | North | Female | 14.75628 | 121.0555 |
| 8 | North | Female | 14.75628 | 121.0555 |
| 9 | North | Female | 14.75628 | 121.0555 |
| 10 | North | Female | 14.74188 | 121.0254 |
| 11 | North | Female | 14.69757 | 120.9935 |
| 12 | North | Female | 14.71154 | 120.98 |
| 1 | North | Male | 14.73111 | 121.065 |
| 2 | North | Male | 14.73212 | 121.0657 |
| 3 | North | Male | 14.67304 | 121.0639 |
| 4 | North | Male | 14.67071 | 121.0541 |
| 5 | North | Male | 14.75693 | 121.0824 |
| 6 | North | Male | 14.74443 | 121.0802 |
| 7 | North | Male | 14.73474 | 121.0208 |
| 1 | South | Female | 14.46394 | 120.9727 |
| 2 | South | Female | 14.46022 | 120.9983 |
| 3 | South | Female | 14.4546 | 120.992 |
| 4 | South | Female | 14.43733 | 120.9858 |
| 5 | South | Female | 14.46103 | 121.0072 |
| 6 | South | Female | 14.45176 | 121.0214 |
| 7 | South | Female | 14.48448 | 121.0309 |
| 8 | South | Female | 14.41149 | 121.0179 |
| 9 | South | Female | 14.43314 | 121.0327 |
| 10 | South | Female | 14.46119 | 121.0512 |
| 11 | South | Female | 14.41149 | 121.0179 |
| 12 | South | Female | 14.41149 | 121.0179 |
| 13 | South | Female | 14.40952 | 121.0329 |
| 14 | South | Female | 14.40952 | 121.0329 |
| 15 | South | Female | 14.40952 | 121.0329 |
| 16 | South | Female | 14.39588 | 121.0329 |
| 17 | South | Female | 14.39588 | 121.0329 |
| 18 | South | Female | 14.39588 | 121.0329 |
| 19 | South | Female | 14.39588 | 121.0329 |
| 20 | South | Female | 14.39588 | 121.0329 |
| 21 | South | Female | 14.39922 | 121.0426 |
| 22 | South | Female | 14.39588 | 121.0329 |
| 23 | South | Female | 14.39588 | 121.0329 |
| 24 | South | Female | 14.39588 | 121.0329 |
| 25 | South | Female | 14.39588 | 121.0329 |
| 26 | South | Female | 14.39119 | 121.0147 |
| 27 | South | Female | 14.39588 | 121.0329 |
| 28 | South | Female | 14.39922 | 121.0426 |
| 1 | South | Male | 14.4546 | 120.992 |
| 2 | South | Male | 14.4546 | 120.992 |
| 3 | South | Male | 14.46022 | 120.9983 |
| 4 | South | Male | 14.43733 | 120.9858 |
| 5 | South | Male | 14.48448 | 121.0309 |
| 6 | South | Male | 14.47028 | 121.0094 |
| 7 | South | Male | 14.49077 | 121.0371 |
| 8 | South | Male | 14.44377 | 121.0349 |
| 9 | South | Male | 14.48543 | 121.007 |
| 10 | South | Male | 14.48543 | 121.007 |
| 11 | South | Male | 14.49114 | 121.0372 |
| 12 | South | Male | 14.49077 | 121.0371 |
| 13 | South | Male | 14.46103 | 121.0072 |
| 14 | South | Male | 14.46119 | 121.0512 |
| 15 | South | Male | 14.41149 | 121.0179 |
| 16 | South | Male | 14.40952 | 121.0329 |
| 17 | South | Male | 14.44035 | 121.0364 |
| 18 | South | Male | 14.37463 | 121.0443 |
| 19 | South | Male | 14.37463 | 121.0443 |
| 20 | South | Male | 14.37463 | 121.0443 |
| 1 | Central | Female | 14.53505 | 120.9821 |
| 2 | Central | Female | 14.53505 | 120.9821 |
| 3 | Central | Female | 14.5131 | 121.0057 |
| 4 | Central | Female | 14.53505 | 120.9821 |
| 5 | Central | Female | 14.53505 | 120.9821 |
| 6 | Central | Female | 14.5131 | 121.0057 |
| 7 | Central | Female | 14.53845 | 120.9947 |
| 8 | Central | Female | 14.53681 | 121.0121 |
| 9 | Central | Female | 14.5131 | 121.0057 |
| 10 | Central | Female | 14.58216 | 120.988 |
| 11 | Central | Female | 14.58216 | 120.988 |
| 12 | Central | Female | 14.58216 | 120.988 |
| 13 | Central | Female | 14.5705 | 120.9845 |
| 14 | Central | Female | 14.58216 | 120.988 |
| 15 | Central | Female | 14.59581 | 121.0221 |
| 16 | Central | Female | 14.57201 | 120.994 |
| 17 | Central | Female | 14.58216 | 120.988 |
| 18 | Central | Female | 14.5705 | 120.9845 |
| 19 | Central | Female | 14.51191 | 121.0429 |
| 20 | Central | Female | 14.51191 | 121.0429 |
| 21 | Central | Female | 14.56858 | 121.0063 |
| 22 | Central | Female | 14.55289 | 121.0212 |
| 23 | Central | Female | 14.55109 | 121.0492 |
| 24 | Central | Female | 14.55109 | 121.0492 |
| 1 | Central | Male | 14.53681 | 121.0121 |
| 2 | Central | Male | 14.53505 | 120.9821 |
| 3 | Central | Male | 14.53505 | 120.9821 |
| 4 | Central | Male | 14.53505 | 120.9821 |
| 5 | Central | Male | 14.5131 | 121.0057 |
| 6 | Central | Male | 14.53505 | 120.9821 |
| 7 | Central | Male | 14.57201 | 120.994 |
| 8 | Central | Male | 14.5705 | 120.9845 |
| 9 | Central | Male | 14.5705 | 120.9845 |
| 10 | Central | Male | 14.58216 | 120.988 |
| 11 | Central | Male | 14.5705 | 120.9845 |
| 12 | Central | Male | 14.51191 | 121.0429 |
| 13 | Central | Male | 14.50747 | 121.055 |
| 14 | Central | Male | 14.52755 | 121.0442 |
| 15 | Central | Male | 14.56858 | 121.0063 |
| 16 | Central | Male | 14.56996 | 121.0165 |
| 17 | Central | Male | 14.55289 | 121.0212 |
| 18 | Central | Male | 14.52755 | 121.0442 |
| 19 | Central | Male | 14.55109 | 121.0492 |
| 20 | Central | Male | 14.55109 | 121.0492 |
| 1 | West | Female | 14.65476 | 120.9872 |
| 2 | West | Female | 14.62313 | 120.9851 |
| 3 | West | Female | 14.62313 | 120.9851 |
| 4 | West | Female | 14.60811 | 120.9769 |
| 5 | West | Female | 14.60811 | 120.9769 |
| 6 | West | Female | 14.62313 | 120.9851 |
| 7 | West | Female | 14.60811 | 120.9769 |
| 8 | West | Female | 14.62313 | 120.9851 |
| 9 | West | Female | 14.61702 | 121.0258 |
| 10 | West | Female | 14.63969 | 121.0272 |
| 11 | West | Female | 14.62948 | 120.9991 |
| 12 | West | Female | 14.61919 | 121.0202 |
| 13 | West | Female | 14.62948 | 120.9991 |
| 14 | West | Female | 14.61702 | 121.0258 |
| 15 | West | Female | 14.65902 | 121.0326 |
| 16 | West | Female | 14.62948 | 120.9991 |
| 17 | West | Female | 14.63649 | 121.004 |
| 18 | West | Female | 14.61775 | 121.0089 |
| 1 | West | Male | 14.66236 | 120.972 |
| 2 | West | Male | 14.65476 | 120.9872 |
| 3 | West | Male | 14.62313 | 120.9851 |
| 4 | West | Male | 14.62313 | 120.9851 |
| 5 | West | Male | 14.60811 | 120.9769 |
| 6 | West | Male | 14.60811 | 120.9769 |
| 7 | West | Male | 14.60811 | 120.9769 |
| 8 | West | Male | 14.61919 | 121.0202 |
| 9 | West | Male | 14.61775 | 121.0089 |
| 10 | West | Male | 14.62586 | 121.0306 |
| 11 | West | Male | 14.64114 | 121.0154 |
| 12 | West | Male | 14.66388 | 121.0213 |
| 1 | East | Female | 14.6491 | 121.0946 |
| 2 | East | Female | 14.65382 | 121.1262 |
| 3 | East | Female | 14.6434 | 121.1159 |
| 4 | East | Female | 14.65125 | 121.1235 |
| 5 | East | Female | 14.64861 | 121.1001 |
| 6 | East | Female | 14.61309 | 121.0465 |
| 7 | East | Female | 14.61238 | 121.0371 |
| 8 | East | Female | 14.6386 | 121.0492 |
| 9 | East | Female | 14.61727 | 121.0692 |
| 10 | East | Female | 14.64169 | 121.0646 |
| 11 | East | Female | 14.62411 | 121.0506 |
| 12 | East | Female | 14.62367 | 121.0478 |
| 13 | East | Female | 14.62411 | 121.0506 |
| 14 | East | Female | 14.62354 | 121.0677 |
| 15 | East | Female | 14.61814 | 121.0629 |
| 16 | East | Female | 14.59661 | 121.028 |
| 17 | East | Female | 14.59273 | 121.0375 |
| 18 | East | Female | 14.5911 | 121.0216 |
| 19 | East | Female | 14.5911 | 121.0216 |
| 1 | East | Male | 14.6514 | 121.106 |
| 2 | East | Male | 14.64861 | 121.1001 |
| 3 | East | Male | 14.64306 | 121.0965 |
| 4 | East | Male | 14.63708 | 121.104 |
| 5 | East | Male | 14.64169 | 121.0646 |
| 6 | East | Male | 14.58635 | 121.06 |
| 7 | East | Male | 14.58635 | 121.06 |
| 8 | East | Male | 14.58635 | 121.06 |
| 9 | East | Male | 14.58831 | 121.0318 |
| 10 | East | Male | 14.59273 | 121.0375 |
| 11 | East | Male | 14.58831 | 121.0318 |
| 12 | East | Male | 14.5911 | 121.0216 |
| 1 | North Manila | Male | 14.61191 | 120.9933 |
| 2 | North Manila | Male | 14.61904 | 120.9986 |
| 3 | North Manila | Male | 14.61904 | 120.9986 |
| 4 | North Manila | Male | 14.61816 | 120.9988 |
| 5 | North Manila | Male | 14.61579 | 120.9956 |
| 6 | North Manila | Male | 14.6072 | 120.9862 |
| 7 | North Manila | Male | 14.61371 | 120.995 |
| 8 | North Manila | Male | 14.61191 | 120.9933 |
| 9 | North Manila | Male | 14.60799 | 120.9867 |
| 10 | North Manila | Male | 14.61294 | 120.9944 |
| 11 | North Manila | Male | 14.61579 | 120.9956 |
| 12 | North Manila | Male | 14.61186 | 120.9953 |
| 13 | North Manila | Male | 14.60599 | 120.9847 |
| 14 | North Manila | Male | 14.61088 | 120.9906 |
| 15 | North Manila | Male | 14.60612 | 120.9849 |
| 1 | North Manila | Female | 14.61057 | 120.9922 |
| 2 | North Manila | Female | 14.60682 | 120.9869 |
| 3 | North Manila | Female | 14.60796 | 120.9852 |
| 4 | North Manila | Female | 14.60796 | 120.9852 |
| 5 | North Manila | Female | 14.60796 | 120.9852 |
| 6 | North Manila | Female | 14.60796 | 120.9852 |
| 7 | North Manila | Female | 14.61464 | 120.9928 |
| 8 | North Manila | Female | 14.61489 | 120.9977 |
| 9 | North Manila | Female | 14.61569 | 120.997 |
| 10 | North Manila | Female | 14.61259 | 120.9917 |
| 11 | North Manila | Female | 14.61579 | 120.9956 |
| 12 | North Manila | Female | 14.61603 | 120.9971 |
| 1 | South Manila | Male | 14.613 | 120.9984 |
| 2 | South Manila | Male | 14.61158 | 120.9954 |
| 3 | South Manila | Male | 14.61075 | 120.9957 |
| 4 | South Manila | Male | 14.61064 | 120.9945 |
| 5 | South Manila | Male | 14.60882 | 120.9954 |
| 6 | South Manila | Male | 14.61239 | 120.9954 |
| 7 | South Manila | Male | 14.60856 | 120.9907 |
| 8 | South Manila | Male | 14.61057 | 120.9957 |
| 9 | South Manila | Male | 14.60888 | 120.993 |
| 1 | South Manila | Female | 14.61334 | 120.9977 |
| 2 | South Manila | Female | 14.613 | 120.9984 |
| 3 | South Manila | Female | 14.61264 | 120.9971 |
| 4 | South Manila | Female | 14.61209 | 120.9974 |
| 5 | South Manila | Female | 14.611 | 120.9953 |
| 6 | South Manila | Female | 14.61097 | 120.9956 |
| 7 | South Manila | Female | 14.61064 | 120.9945 |
| 8 | South Manila | Female | 14.6098 | 120.9943 |
| 9 | South Manila | Female | 14.60915 | 120.9958 |
| 10 | South Manila | Female | 14.60895 | 120.9956 |
| 11 | South Manila | Female | 14.60863 | 120.9955 |
| 12 | South Manila | Female | 14.61158 | 120.9954 |
| 13 | South Manila | Female | 14.61042 | 120.9969 |
| 14 | South Manila | Female | 14.61042 | 120.9969 |
| 15 | South Manila | Female | 14.61012 | 120.9971 |
| 16 | South Manila | Female | 14.61012 | 120.9971 |

**Table S2** Characteristics of satellite data obtained from Google Earth Engine

| **Data** | **Product ID** | **Bands** | **Spatial resolution** | **Temporal resolution** | **Unit** | **Source** |
| --- | --- | --- | --- | --- | --- | --- |
| Precipitation | NASA/GPM_L3/IMERG_V06 | precipitationCal | 11132 m | Hourly | mm/h | (Huffman, et al., 2019) |
| Land Surface Temperature | MODIS/006/MOD11A1 | LST_Day_1km  LST_Night_1km | 1000 m | Daily | K | (Wan, et al., 2021; Wan, et al., 2015) |
|  | MODIS/006/MYD11A1 | LST_Day_1km  LST_Night_1km | 1000 m | Daily | K |  |
| Dew-point temperature | ECMWF/ERA5_LAND/HOURLY | dewpoint_temperature_2m | 11132 m | Hourly | ^o^C | (Muñoz Sabater, 2019) |
| Air Temperature | ECMWF/ERA5_LAND/HOURLY | temperature_2m | 11132 m | Hourly | ^o^C |  |
| Wind speed | ECMWF/ERA5_LAND/HOURLY | u_component_of_wind_10m | 11132 m | Hourly | m/s |  |
|  | ECMWF/ERA5_LAND/HOURLY | v_component_of_wind_10m | 11132 m | Hourly | m/s |  |

**Table S3** Environmental variables mean value per sampling region/population.

| Population | Landscape variables | | | | | | | | | Climatic varibles | | | | |
| --- | --- | --- | --- | --- | --- | --- | --- | --- | --- | --- | --- | --- | --- | --- |
|  | Agriculture (%) | Grass land (%) | Forest (%) | Water bodies (%) | Open space (%) | Park and recreation (%) | Residential area (%) | Building (%) | NoD (%) | Air temperature (^o^C) | Precipitation (mm/h) | Eastward Wind (m/s) | Northward Wind (m/s) | Relative Humidity (%) |
| F_Central | 0 | 0.06 | 0.24 | 1.37 | 8.62 | 5.88 | 29.04 | 49.49 | 5.3 | 26.36 | 595.87 | 0.13 | 0.26 | 81.99 |
| F_East | 0.83 | 0.4 | 0.08 | 0.67 | 0.7 | 0.31 | 66.18 | 29.05 | 1.78 | 26.66 | 679.66 | 1.06 | 0.83 | 85.35 |
| F_North | 0.93 | 0.44 | 7.47 | 4.31 | 9.29 | 0.94 | 64.17 | 11.61 | 0.84 | 27.35 | 517.53 | -0.34 | 0.02 | 79.96 |
| F_South | 1.11 | 0.1 | 0.15 | 0.7 | 7.65 | 2.45 | 68.95 | 18.69 | 0.2 | 26.61 | 477.05 | -0.6 | 0.2 | 80.94 |
| F_West | 0 | 0 | 0 | 1.26 | 2.2 | 0.96 | 59.06 | 31.12 | 5.4 | 26.74 | 634.3 | 0.58 | 0.42 | 82.93 |
| M_Central | 0.44 | 0.07 | 0.18 | 1.43 | 10.3 | 4.59 | 34.89 | 44.25 | 3.85 | 26.25 | 560.78 | -0.11 | 0.05 | 81.89 |
| M_East | 2.23 | 0.86 | 0.05 | 0.92 | 3.19 | 0.54 | 64.08 | 27 | 1.13 | 26.05 | 506.56 | -0.07 | 0.2 | 82.03 |
| M_North | 0.59 | 0.9 | 12.44 | 4.08 | 4 | 0.12 | 70.53 | 7.11 | 0.23 | 27.62 | 529.99 | 0.27 | 0.35 | 84.68 |
| M_South | 0.9 | 0.23 | 0.24 | 0.18 | 9.23 | 0.68 | 71.9 | 16.32 | 0.32 | 27.57 | 528.18 | 0.74 | 0.82 | 80.5 |
| M_West | 0 | 0 | 0 | 1.25 | 1.34 | 0.89 | 59.84 | 31.69 | 4.99 | 26.98 | 508.7 | 0.42 | 0.62 | 82.11 |
| F_Manila_North | 0 | 0 | 0 | 1.7 | 0.12 | 1.47 | 52.3 | 37.18 | 7.23 | 27.25 | 722.52 | 0.58 | 0.36 | 84.32 |
| F_Manila_South | 0 | 0 | 0 | 2.81 | 0.14 | 0.16 | 65.22 | 25.51 | 6.16 | 27.13 | 802.42 | 1.01 | 0.69 | 84.55 |
| M_Manila_North | 0 | 0 | 0 | 2.26 | 0.12 | 0.35 | 64.21 | 26.34 | 6.72 | 27.25 | 722.52 | 0.58 | 0.36 | 84.32 |
| M_Manila_South | 0 | 0 | 0 | 2.81 | 0.14 | 0.16 | 65.22 | 25.51 | 6.16 | 27.07 | 838.74 | 1.21 | 0.84 | 84.65 |

**Table S4** Correlation matrix between environmental variables, with correlation coefficient shown in top right, and P-values in bottom left.

|  | Agricultural | Grass land | Forest | water bodies | Open space | Park and recriation | Residential area | building | Air temperature | Precipitation | Eastward Wind | Northward Wind | Relative Humidity |
| --- | --- | --- | --- | --- | --- | --- | --- | --- | --- | --- | --- | --- | --- |
| Agricultural |  | 0 | 0.0189 | 0.2333 | 0.0327 | 0.9682 | 0.116 | 0.0778 | 0.6715 | 0.0036 | 0.0703 | 0.1177 | 0.116 |
| Grass land | 0.87 |  | 0.0003 | 0.6622 | 0.0206 | 0.6736 | 0.1689 | 0.0996 | 0.9752 | 0.0227 | 0.1122 | 0.1239 | 0.4501 |
| Forest | 0.62 | 0.83 |  | 0.938 | 0.0003 | 0.6001 | 0.4071 | 0.2 | 0.6391 | 0.0603 | 0.058 | 0.0699 | 0.1353 |
| water bodies | -0.34 | -0.13 | 0.02 |  | 0.6036 | 0.321 | 0.6855 | 0.3612 | 0.0976 | 0.1481 | 0.9104 | 0.4675 | 0.4173 |
| Open space | 0.57 | 0.61 | 0.82 | -0.15 |  | 0.0919 | 0.8988 | 0.6685 | 0.5412 | 0.0103 | 0.0107 | 0.028 | 0.0025 |
| Park and recriation | 0.01 | -0.12 | 0.15 | -0.29 | 0.47 |  | 0.0108 | 0.0292 | 0.0923 | 0.229 | 0.025 | 0.041 | 0.0066 |
| Residential area | 0.44 | 0.39 | 0.24 | -0.12 | -0.04 | -0.66 |  | 0.0001 | 0.0715 | 0.7474 | 0.3154 | 0.1784 | 0.492 |
| building | -0.49 | -0.46 | -0.36 | -0.26 | -0.13 | 0.58 | -0.85 |  | 0.0153 | 0.5018 | 0.9821 | 0.8988 | 0.9345 |
| Air temperature | -0.12 | 0.01 | 0.14 | 0.46 | -0.18 | -0.47 | 0.5 | -0.63 |  | 0.4586 | 0.2854 | 0.3732 | 0.5987 |
| Precipitation | -0.72 | -0.6 | -0.51 | 0.41 | -0.66 | -0.34 | -0.09 | 0.2 | 0.22 |  | 0.0015 | 0.0329 | 0.0101 |
| Eastward Wind | -0.5 | -0.44 | -0.52 | -0.03 | -0.66 | -0.59 | 0.29 | -0.01 | 0.31 | 0.76 |  | 0 | 0.0082 |
| Northward Wind | -0.44 | -0.43 | -0.5 | -0.21 | -0.58 | -0.55 | 0.38 | -0.04 | 0.26 | 0.57 | 0.95 |  | 0.024 |
| Relative Humidity | -0.44 | -0.22 | -0.42 | 0.24 | -0.74 | -0.69 | 0.2 | 0.02 | 0.15 | 0.66 | 0.67 | 0.6 |  |

**Table S5** Association between non-synonymous SNPs and environmental variables based on the variable selection analysis.

1. **Male populations**

| **Chr** | **Position** | **Environmental variable** | **P value** | **F** | **Environmental variable** | **P value** | **F** | **Environmental variable** | **P value** | **F** |
| --- | --- | --- | --- | --- | --- | --- | --- | --- | --- | --- |
| 1 | 18310770 | building | 0.005 | 95.8344 |  |  |  |  |  |  |
| 1 | 27329590 |  |  |  |  |  |  |  |  |  |
| 1 | 50256460 | building | 0.03 | 16.7316 |  |  |  |  |  |  |
| 1 | 52006475 | Water bodies | 0.015 | 12.6444 |  |  |  |  |  |  |
| 1 | 52855173 |  |  |  |  |  |  |  |  |  |
| 1 | 82513659 |  |  |  |  |  |  |  |  |  |
| 1 | 97369379 |  |  |  |  |  |  |  |  |  |
| 1 | 97369381 |  |  |  |  |  |  |  |  |  |
| 1 | 128938627 | precipitation | 0.03 | 16.8819 |  |  |  |  |  |  |
| 1 | 148625328 | Water bodies | 0.02 | 11.4357 |  |  |  |  |  |  |
| 1 | 162884116 | building | 0.025 | 31.2363 |  |  |  |  |  |  |
| 1 | 173645184 | building | 0.01 | 8.6325 |  |  |  |  |  |  |
| 1 | 192629059 |  |  |  |  |  |  |  |  |  |
| 1 | 193102226 |  |  |  |  |  |  |  |  |  |
| 1 | 200315828 |  |  |  |  |  |  |  |  |  |
| 1 | 207947979 |  |  |  |  |  |  |  |  |  |
| 1 | 224425805 |  |  |  |  |  |  |  |  |  |
| 1 | 237699392 |  |  |  |  |  |  |  |  |  |
| 1 | 237699398 |  |  |  |  |  |  |  |  |  |
| 1 | 237699409 |  |  |  |  |  |  |  |  |  |
| 1 | 237699416 |  |  |  |  |  |  |  |  |  |
| 1 | 252278611 |  |  |  |  |  |  |  |  |  |
| 1 | 256971608 | park and recreation | 0.01 | 5.4847 |  |  |  |  |  |  |
| 1 | 273779321 |  |  |  |  |  |  |  |  |  |
| 2 | 24745865 |  |  |  |  |  |  |  |  |  |
| 2 | 71505075 | forest | 0.005 | 1.7165 |  |  |  |  |  |  |
| 2 | 75969638 | park and recreation | 0.005 | 227.4445 |  |  |  |  |  |  |
| 2 | 93211523 |  |  |  |  |  |  |  |  |  |
| 2 | 101967349 | air temperature | 0.03 | 29.0932 | open space | 0.035 | 7.8367 |  |  |  |
| 2 | 103173274 |  |  |  |  |  |  |  |  |  |
| 2 | 105609353 | air temperature | 0.02 | 8.0605 | building | 0.02 | 8.0851 |  |  |  |
| 2 | 107987295 | air temperature | 0.035 | 8.4563 |  |  |  |  |  |  |
| 2 | 107987297 |  |  |  |  |  |  |  |  |  |
| 2 | 120537772 |  |  |  |  |  |  |  |  |  |
| 2 | 143355555 | park and recreation | 0.03 | 22.2454 |  |  |  |  |  |  |
| 2 | 168938157 |  |  |  |  |  |  |  |  |  |
| 2 | 184038539 | Water bodies | 0.04 | 3.183 | precipitation | 0.035 | 3.4019 |  |  |  |
| 2 | 205470997 |  |  |  |  |  |  |  |  |  |
| 2 | 206310895 |  |  |  |  |  |  |  |  |  |
| 2 | 215925135 | park and recreation | 0.01 | 42.7132 |  |  |  |  |  |  |
| 2 | 218578639 | air temperature | 0.025 | 10.193 | park and recreation | 0.005 | 21.1458 |  |  |  |
| 2 | 255763248 | air temperature | 0.045 | 4.5793 | agriculture | 0.01 | 11.4901 | building | 0.005 | 454.1984 |
| 2 | 273356906 | building | 0.015 | 5.9391 |  |  |  |  |  |  |
| 2 | 286998675 |  |  |  |  |  |  |  |  |  |
| 2 | 295318921 |  |  |  |  |  |  |  |  |  |
| 2 | 295318923 |  |  |  |  |  |  |  |  |  |
| 2 | 295318959 |  |  |  |  |  |  |  |  |  |
| 2 | 295318962 |  |  |  |  |  |  |  |  |  |
| 2 | 403078007 | northward wind | 0.035 | 3.6141 |  |  |  |  |  |  |
| 2 | 420390053 |  |  |  |  |  |  |  |  |  |
| 2 | 425994458 |  |  |  |  |  |  |  |  |  |
| 2 | 446238854 |  |  |  |  |  |  |  |  |  |
| 3 | 284390 |  |  |  |  |  |  |  |  |  |
| 3 | 10648043 |  |  |  |  |  |  |  |  |  |
| 3 | 43194058 |  |  |  |  |  |  |  |  |  |
| 3 | 56658773 | forest | 0.03 | 3.7616 |  |  |  |  |  |  |
| 3 | 77208493 | precipitation | 0.03 | 6.839 |  |  |  |  |  |  |
| 3 | 78311617 | forest | 0.01 | 10.8683 | open space | 0.005 | 19.5269 |  |  |  |
| 3 | 86903052 |  |  |  |  |  |  |  |  |  |
| 3 | 88143715 |  |  |  |  |  |  |  |  |  |
| 3 | 93050362 |  |  |  |  |  |  |  |  |  |
| 3 | 119325294 | forest | 0.035 | 2.4907 |  |  |  |  |  |  |
| 3 | 174163301 |  |  |  |  |  |  |  |  |  |
| 3 | 180389967 |  |  |  |  |  |  |  |  |  |
| 3 | 199350760 | park and recreation | 0.025 | 197.1844 |  |  |  |  |  |  |
| 3 | 204352142 |  |  |  |  |  |  |  |  |  |
| 3 | 205982858 |  |  |  |  |  |  |  |  |  |
| 3 | 205982861 |  |  |  |  |  |  |  |  |  |
| 3 | 205982862 |  |  |  |  |  |  |  |  |  |
| 3 | 246681869 |  |  |  |  |  |  |  |  |  |
| 3 | 292219868 | park and recreation | 0.01 | 230.2836 |  |  |  |  |  |  |
| 3 | 295070690 | building | 0.03 | 5.8233 |  |  |  |  |  |  |
| 3 | 309404428 | forest | 0.035 | 4.255 |  |  |  |  |  |  |
| 3 | 385022347 |  |  |  |  |  |  |  |  |  |
| 3 | 406625999 |  |  |  |  |  |  |  |  |  |
| 3 | 409736616 | park and recreation | 0.02 | 65.6297 |  |  |  |  |  |  |

1. **Female populations**

| **Chr** | Position | Environmental variable | P value | F | Environmental variable | P value | F | Environmental variable | P value | F |
| --- | --- | --- | --- | --- | --- | --- | --- | --- | --- | --- |
| 1 | 18310770 | agriculture | 0.005 | 9.3556 | air temperature | 0.02 | 9.6973 |  |  |  |
| 1 | 27329590 | open space | 0.03 | 8.0996 |  |  |  |  |  |  |
| 1 | 50256460 |  |  |  |  |  |  |  |  |  |
| 1 | 52006475 | air temperature | 0.03 | 6.5863 |  |  |  |  |  |  |
| 1 | 52855173 | forest | 0.025 | 2.909 |  |  |  |  |  |  |
| 1 | 82513659 | forest | 0.04 | 2.909 |  |  |  |  |  |  |
| 1 | 97369379 | park and recreation | 0.015 | 17.2423 | grassland | 0.01 | 18.7039 | Residential area | 0.045 | 5.7033 |
| 1 | 97369381 | park and recreation | 0.015 | 17.3789 | grassland | 0.02 | 15.3293 | Residential area | 0.04 | 6.514 |
| 1 | 128938627 |  |  |  |  |  |  |  |  |  |
| 1 | 148625328 | forest | 0.01 | 9.2416 | open space | 0.005 | 35.1705 |  |  |  |
| 1 | 162884116 |  |  |  |  |  |  |  |  |  |
| 1 | 173645184 | building | 0.02 | 8.4223 |  |  |  |  |  |  |
| 1 | 192629059 | air temperature | 0.015 | 5.2209 |  |  |  |  |  |  |
| 1 | 193102226 | Residential area | 0.005 | 34.1569 |  |  |  |  |  |  |
| 1 | 200315828 |  |  |  |  |  |  |  |  |  |
| 1 | 207947979 | air temperature | 0.005 | 3.7771 | Residential area | 0.025 | 17.1299 |  |  |  |
| 1 | 224425805 | building | 0.015 | 7.7924 | park and recreation | 0.02 | 16.63 |  |  |  |
| 1 | 237699392 |  |  |  |  |  |  |  |  |  |
| 1 | 237699398 |  |  |  |  |  |  |  |  |  |
| 1 | 237699409 |  |  |  |  |  |  |  |  |  |
| 1 | 237699416 |  |  |  |  |  |  |  |  |  |
| 1 | 252278611 | air temperature | 0.02 | 3.934 | Residential area | 0.02 | 12.952 |  |  |  |
| 1 | 256971608 |  |  |  |  |  |  |  |  |  |
| 1 | 273779321 | air temperature | 0.015 | 3.6848 | Residential area | 0.01 | 16.6121 |  |  |  |
| 2 | 24745865 | relative humidity | 0.015 | 11.8158 | park and recreation | 0.015 | 5.9931 |  |  |  |
| 2 | 71505075 | building | 0.01 | 14.6953 |  |  |  |  |  |  |
| 2 | 75969638 |  |  |  |  |  |  |  |  |  |
| 2 | 93211523 |  |  |  |  |  |  |  |  |  |
| 2 | 101967349 |  |  |  |  |  |  |  |  |  |
| 2 | 103173274 | air temperature | 0.01 | 3.9151 | Residential area | 0.02 | 14.6252 |  |  |  |
| 2 | 105609353 | northward wind | 0.015 | 17.0606 |  |  |  |  |  |  |
| 2 | 107987295 | air temperature | 0.005 | 4.7071 | Residential area | 0.01 | 20.3297 |  |  |  |
| 2 | 107987297 |  |  |  |  |  |  |  |  |  |
| 2 | 120537772 | building | 0.035 | 6.968 | park and recreation | 0.04 | 11.3459 | eastward wind | 0.04 | 10.511 |
| 2 | 143355555 | air temperature | 0.005 | 4.0389 | Residential area | 0.005 | 21.2489 |  |  |  |
| 2 | 168938157 | air temperature | 0.005 | 3.8955 | Residential area | 0.01 | 17.9494 |  |  |  |
| 2 | 184038539 |  |  |  |  |  |  |  |  |  |
| 2 | 205470997 | northward wind | 0.015 | 12.7687 |  |  |  |  |  |  |
| 2 | 206310895 | forest | 0.02 | 1.4065 |  |  |  |  |  |  |
| 2 | 215925135 | air temperature | 0.015 | 3.7146 | Residential area | 0.02 | 14.5826 |  |  |  |
| 2 | 218578639 | air temperature | 0.025 | 8.1371 |  |  |  |  |  |  |
| 2 | 255763248 | air temperature | 0.005 | 4.1124 | Residential area | 0.005 | 17.6404 |  |  |  |
| 2 | 273356906 |  |  |  |  |  |  |  |  |  |
| 2 | 286998675 | Residential area | 0.025 | 28.1706 |  |  |  |  |  |  |
| 2 | 295318921 |  |  |  |  |  |  |  |  |  |
| 2 | 295318923 | Water bodies | 0.04 | 5.9846 |  |  |  |  |  |  |
| 2 | 295318959 |  |  |  |  |  |  |  |  |  |
| 2 | 295318962 |  |  |  |  |  |  |  |  |  |
| 2 | 403078007 | air temperature | 0.01 | 12.3961 |  |  |  |  |  |  |
| 2 | 420390053 |  |  |  |  |  |  |  |  |  |
| 2 | 425994458 | Water bodies | 0.01 | 12.3565 |  |  |  |  |  |  |
| 2 | 446238854 | forest | 0.005 | 7.0141 |  |  |  |  |  |  |
| 3 | 284390 | Residential area | 0.02 | 9.7238 |  |  |  |  |  |  |
| 3 | 10648043 |  |  |  |  |  |  |  |  |  |
| 3 | 43194058 | open space | 0.005 | 18.0351 | eastward wind | 0.015 | 4.8174 |  |  |  |
| 3 | 56658773 | forest | 0.025 | 1.8015 |  |  |  |  |  |  |
| 3 | 77208493 |  |  |  |  |  |  |  |  |  |
| 3 | 78311617 | forest | 0.005 | 8.0201 |  |  |  |  |  |  |
| 3 | 86903052 |  |  |  |  |  |  |  |  |  |
| 3 | 88143715 | eastward wind | 0.025 | 8.6655 |  |  |  |  |  |  |
| 3 | 93050362 | grassland | 0.03 | 5.8911 |  |  |  |  |  |  |
| 3 | 119325294 | building | 0.015 | 15.5978 | grassland | 0.035 | 9.9344 |  |  |  |
| 3 | 174163301 | grassland | 0.03 | 6.7404 | forest | 0.015 | 15.6241 |  |  |  |
| 3 | 180389967 | air temperature | 0.005 | 4.5758 | Residential area | 0.01 | 20.5155 |  |  |  |
| 3 | 199350760 |  |  |  |  |  |  |  |  |  |
| 3 | 204352142 | open space | 0.025 | 7.7386 | relative humidity | 0.02 | 16.5487 | agriculture | 0.01 | 12.0581 |
| 3 | 205982858 | eastward wind | 0.005 | 6.8766 | northward wind | 0.015 | 33.789 |  |  |  |
| 3 | 205982861 | eastward wind | 0.02 | 6.8766 | northward wind | 0.005 | 33.789 |  |  |  |
| 3 | 205982862 | eastward wind | 0.01 | 6.8766 | northward wind | 0.005 | 33.789 |  |  |  |
| 3 | 246681869 | air temperature | 0.005 | 4.2203 | Residential area | 0.005 | 17.8071 |  |  |  |
| 3 | 292219868 | air temperature | 0.02 | 5.2631 | park and recreation | 0.015 | 8.9849 |  |  |  |
| 3 | 295070690 |  |  |  |  |  |  |  |  |  |
| 3 | 309404428 |  |  |  |  |  |  |  |  |  |
| 3 | 385022347 | air temperature | 0.015 | 3.9128 | park and recreation | 0.02 | 13.5069 |  |  |  |
| 3 | 406625999 |  |  |  |  |  |  |  |  |  |
| 3 | 409736616 | air temperature | 0.005 | 3.7124 | Residential area | 0.01 | 21.9633 |  |  |  |

**Table S6** Within-Gene or near-gene SNPs among the putative outlier identified using PCA-, Bayesian- and Fst-based empirical detection

|  | **Chromosome** | **Position** | **Gene/Protein** | **Percent identity** | **Function** |
| --- | --- | --- | --- | --- | --- |
| 1 | 1 | 27329590 | protein lava lamp isoform X1 | 100.00% | function not determined |
| 2 | 1 | 50256460 | AP-like endonuclease reverse transcriptase | 40.85% | endonuclease activity, RNA-directed DNA polymerase activity |
| 3 | 1 | 52006475 | coiled-coil domain-containing protein 158-like | 59.13% | function not determined |
| 4 | 1 | 52855173 | uncharacterized protein K02A2.6-like | 76.35% | nucleic acid binding, DNA integration |
| 5 | 1 | 82513659 | uncharacterized protein LOC110675653 | 100.00% | function not determined |
| 6 | 1 | 128938627 | hypothetical protein RP20_CCG007352 | 67.90% | Reverse transcriptase domain-containing protein |
| 7 | 1 | 162884116 | AAEL017247-PA | 86.67% | function not determined |
| 8 | 1 | 173645184 | hypothetical protein RP20_CCG005523 | 63.45% | nucleic acid binding, zinc ion binding |
| 9 | 1 | 207947979 | gag-like protein | 35.83% | function not determined |
| 10 | 1 | 224425805 | hypothetical protein RP20_CCG027089 | 46.88% | nucleic acid binding, zinc ion binding, DNA biosynthetic process |
| 11 | 1 | 252278611 | zinc finger MYM-type protein 1-like | 65.83% | protein dimerization activity |
| 12 | 1 | 256971608 | hypothetical protein RP20_CCG023927 | 53.43% | function not determined |
| 13 | 1 | 273779321 | uncharacterized protein LOC109429518 | 54.10% | function not determined |
| 14 | 2 | 71505075 | uncharacterized protein LOC119766262 | 46.24% | function not determined |
| 15 | 2 | 93211523 | uncharacterized protein LOC109432264 | 87.73% | function not determined |
| 16 | 2 | 101967349 | uncharacterized protein K02A2.6-like | 67.35% | nucleic acid binding, DNA integration |
| 17 | 2 | 107987295 | AAEL017170-PA | 54.10% | metal ion binding |
| 18 | 2 | 107987297 | AAEL017170-PA | 55.00% | metal ion binding |
| 19 | 2 | 120537772 | PREDICTED: uncharacterized protein LOC108367860 isoform X1 | 60.00% | function not determined |
| 20 | 2 | 168938157 | hypothetical protein RP20_CCG005656 | 82.77% | DNA biosynthetic process |
| 21 | 2 | 205470997 | uncharacterized protein LOC119613850 | 45.00% | function not determined |
| 22 | 2 | 206310895 | uncharacterized protein K02A2.6-like | 91.49% | nucleic acid binding, DNA integration |
| 23 | 2 | 255763248 | hypothetical protein RP20_CCG001022 | 62.11% | DNA polymerase complex,DNA-directed DNA polymerase activity, nucleic acid binding, DNA integration |
| 24 | 2 | 273356906 | uncharacterized protein LOC110676845 | 61.36% | function not determined |
| 25 | 2 | 286998675 | uncharacterized protein LOC115270316 | 62.23% | function not determined |
| 26 | 2 | 295318921 | uncharacterized protein Dwil_GK26988 | 98.48% | DNA biosynthetic process |
| 27 | 2 | 295318923 | uncharacterized protein Dwil_GK26988 | 98.48% | DNA biosynthetic process |
| 28 | 2 | 295318959 | uncharacterized protein Dwil_GK26988 | 98.57% | DNA biosynthetic process |
| 29 | 2 | 295318962 | uncharacterized protein Dwil_GK26988 | 98.58% | DNA biosynthetic process |
| 30 | 2 | 403078007 | reverse transcriptase-like protein | 90.37% | nucleic acid binding, RNA-directed DNA polymerase activity, zinc ion binding |
| 31 | 2 | 425994458 | uncharacterized protein LOC115264297 | 35.61% | function not determined |
| 32 | 2 | 446238854 | AAEL010489-PA [Aedes aegypti], transcriptional regulator ATRX homolog isoform X1 | 100.00% | ATP binding, ATP hydrolysis activity, ATP-dependent chromatin remodeler activity, DNA binding |
| 33 | 3 | 284390 | uncharacterized protein LOC119770245 | 43.11% | function not determined |
| 34 | 3 | 10648043 | tRNA (cytosine(34)-C(5))-methyltransferase | 100.00% | function not determined |
| 35 | 3 | 77208493 | uncharacterized protein Dwil_GK26988 | 74.21% | DNA biosynthetic process |
| 36 | 3 | 86903052 | uncharacterized protein LOC119768603 | 65.29% | function not determined |
| 37 | 3 | 119325294 | reverse transcriptase | 51.53% | function not determined |
| 38 | 3 | 180389967 | hypothetical protein RP20_CCG005656 | 61.07% | DNA biosynthetic process |
| 39 | 3 | 199350760 | uncharacterized protein LOC115263935 | 56.98% | function not determined |
| 40 | 3 | 204352142 | syntaxin-18 isoform X1 | 97.99% | function not determined |
| 41 | 3 | 205982858 | hypothetical protein RP20_CCG000451 | 50.88% | DNA polymerase complex,DNA-directed DNA polymerase activity,metal ion binding, nucleic acid binding, DNA integration |
| 42 | 3 | 205982861 | hypothetical protein RP20_CCG000451 | 52.04% | DNA polymerase complex,DNA-directed DNA polymerase activity,metal ion binding, nucleic acid binding, DNA integration |
| 43 | 3 | 205982862 | hypothetical protein RP20_CCG000451 | 52.04% | DNA polymerase complex,DNA-directed DNA polymerase activity,metal ion binding, nucleic acid binding, DNA integration |
| 44 | 3 | 246681869 | hypothetical protein RP20_CCG012321 | 61.11% | nucleic acid binding,DNA integration |
| 45 | 3 | 292219868 | uncharacterized protein LOC125768526 isoform X2 | 63.96% | function not determined |
| 46 | 3 | 295070690 | stress response protein NST1-like , nucleoporin GLE1-like | 70.18% | Mrna binding, Mrna processing, mrna transport, nuclear-transcribed Mrna catabolic process, nonsense-mediated decay, regulation of translation, RNA splicing |
| 47 | 3 | 309404428 | fatty acyl-CoA reductase 1 | 99.03% | Catalyzes the reduction of fatty acyl-CoA to fatty alcohols. |
| 48 | 3 | 385022347 | uncharacterized protein LOC115267902 | 78.95% | function not determined |
| 49 | 3 | 409736616 | uncharacterized protein LOC115259497 | 74.73% | function not determined |
